# Integrating RNA-seq and ChIP-seq data to characterize long non-coding RNAs in *Drosophila melanogaster*

**DOI:** 10.1186/s12864-016-2457-0

**Published:** 2016-03-11

**Authors:** Mei-Ju May Chen, Li-Kai Chen, Yu-Shing Lai, Yu-Yu Lin, Dung-Chi Wu, Yi-An Tung, Kwei-Yan Liu, Hsueh-Tzu Shih, Yi-Jyun Chen, Yan-Liang Lin, Li-Ting Ma, Jian-Long Huang, Po-Chun Wu, Ming-Yi Hong, Fang-Hua Chu, June-Tai Wu, Wen-Hsiung Li, Chien-Yu Chen

**Affiliations:** Genome and Systems Biology Degree Program, National Taiwan University and Academia Sinica, Taipei, 106 Taiwan; Institute of Molecular Medicine, College of Medicine, National Taiwan University, Taipei, 100 Taiwan; Department of Bio-Industrial Mechatronics Engineering, National Taiwan University, Taipei, 106 Taiwan; Graduate Institute of Biomedical Electronics and Bioinformatics, National Taiwan University, Taipei, 106 Taiwan; School of Forestry and Resource Conservation, National Taiwan University, Taipei, 106 Taiwan; Department of Medical Research, National Taiwan University Hospital, Taipei, 100 Taiwan; Research Center for Developmental Biology and Regenerative Medicine, National Taiwan University, Taipei, 100 Taiwan; Biodiversity Research Center, Academia Sinica, Taipei, 115 Taiwan; Department of Ecology and Evolution, University of Chicago, Chicago, IL 60637 USA; Center for Systems Biology, National Taiwan University, Taipei, 106 Taiwan

**Keywords:** Long non-coding RNA, Active transcription, ChIP-seq, RNA-seq, *Drosophila melanogaster*

## Abstract

**Background:**

Recent advances in sequencing technology have opened a new era in RNA studies. Novel types of RNAs such as long non-coding RNAs (lncRNAs) have been discovered by transcriptomic sequencing and some lncRNAs have been found to play essential roles in biological processes. However, only limited information is available for lncRNAs in *Drosophila melanogaster*, an important model organism. Therefore, the characterization of lncRNAs and identification of new lncRNAs in *D. melanogaster* is an important area of research. Moreover, there is an increasing interest in the use of ChIP-seq data (H3K4me3, H3K36me3 and Pol II) to detect signatures of active transcription for reported lncRNAs.

**Results:**

We have developed a computational approach to identify new lncRNAs from two tissue-specific RNA-seq datasets using the poly(A)-enriched and the ribo-zero method, respectively. In our results, we identified 462 novel lncRNA transcripts, which we combined with 4137 previously published lncRNA transcripts into a curated dataset. We then utilized 61 RNA-seq and 32 ChIP-seq datasets to improve the annotation of the curated lncRNAs with regards to transcriptional direction, exon regions, classification, expression in the brain, possession of a poly(A) tail, and presence of conventional chromatin signatures. Furthermore, we used 30 time-course RNA-seq datasets and 32 ChIP-seq datasets to investigate whether the lncRNAs reported by RNA-seq have active transcription signatures. The results showed that more than half of the reported lncRNAs did not have chromatin signatures related to active transcription. To clarify this issue, we conducted RT-qPCR experiments and found that ~95.24 % of the selected lncRNAs were truly transcribed, regardless of whether they were associated with active chromatin signatures or not.

**Conclusions:**

In this study, we discovered a large number of novel lncRNAs, which suggests that many remain to be identified in *D. melanogaster*. For the lncRNAs that are known, we improved their characterization by integrating a large number of sequencing datasets (93 sets in total) from multiple sources (lncRNAs, RNA-seq and ChIP-seq). The RT-qPCR experiments demonstrated that RNA-seq is a reliable platform to discover lncRNAs. This set of curated lncRNAs with improved annotations can serve as an important resource for investigating the function of lncRNAs in *D. melanogaster*.

**Electronic supplementary material:**

The online version of this article (doi:10.1186/s12864-016-2457-0) contains supplementary material, which is available to authorized users.

## Background

A RNA sequence is classified as a long non-coding RNA (lncRNA) if it lacks coding potential and has a length >200 base pairs (bp) [1]. Many lncRNAs have been shown to play a role in development and diseases [2, 3]. Additionally, studies on mouse and human have reported that lncRNA genes are similar to protein coding genes in that they contain promoters and transcribed regions. Upon transcription, these regions will have active chromatin signatures such as the tri-methylation of histone H3 lysine 4 (H3K4me3) and the tri-methylation of histone H3 lysine 36 (H3K36me3) [4–6]. It has also been revealed that lncRNA expression may require specific binding of transcription factors to drive RNA polymerase II (Pol II)-mediated transcription [7–9].

In *Drosophila melanogaster*, some lncRNAs have been observed to regulate developmental processes. For example, roX1 and roX2 recruit the MSL (male specific lethal) chromatin remodelling complex to genes on the male X chromosome, but not the autosomes or the female X chromosomes, to increase the acetylation of histone H4K16 [10]. This regulation can coordinate the dosage compensation required for male development. While the functionality of some lncRNAs in fruit fly was known, some lncRNAs have not yet been functionally characterized.

Transcriptional direction is an important characteristic in lncRNAs. The transcripts of lncRNAs are able to disrupt the transcription of coding genes, a phenomenon known as convergent transcription in which the transcriptional direction of the lncRNA and the mRNA are head-to-head against each other [11, 12]. Conversely, for divergent transcription, the lncRNA/mRNA gene pair exhibit coordinated changes in transcription [13]. In this regard, the direction of lncRNA transcription is an important feature to be annotated. Another essential characteristic is the exon regions.

To assess the current state of lncRNA annotation in the fruit fly, we collected known *Drosophila melanogaster* lncRNAs from databases and the literature, and then used strand-specific RNA-seq datasets (Table 1) to add to the characterization of the annotations. The collected lncRNAs contained approximately 3300 genes. To investigate whether many more lncRNAs could be discovered, we obtained additional RNA-seq datasets from the brain (Table 1). We selected the brain, instead of the whole body, because many lncRNAs were tissue-specific according to lncRNA studies in mammals [14]. Also, the brain is important for studying neuron-related diseases. Since some lncRNAs may not contain poly(A) tails, both poly(A)-enriched and ribo-zero libraries were constructed in this study. For the purpose of discovering novel lncRNAs, we developed a reference-based assembly approach to identify potential lncRNA transcripts.

**Table 1:** Summary statistics of datasets used in study

Platforms	Types	Total number of datasets	Experimental condition	Number of datasets
Public RNA-seq (59 in total)	Paired-end without strand-specific	30	Time course/whole body	30
	Paired-end with strand-specific	29	Tissue/head	9
			Tissue/ovary	2
			Tissue/accessory glands	1
			Tissue/testis	1
			Tissue/carcass	4
			Tissue/digestive system	4
			Tissue/CNS	2
			Tissue/fat body	3
			Tissue/imaginal discs	1
			Tissue/salivary glands	2
In-house RNA-seq (2 in total)	Paired-end with poly(A)-enriched	1	Tissue/brain	1
	Paired-end with ribo-zero	1	Tissue/brain	1
ChIP-seq (32 in total)	H3K36me3	3	Embryos	1
			Larvae	1
			Mixed Adult	1
	H3K4me3	14	Embryos	7
			Larvae	3
			Pupae	1
			Adult Female	1
			Adult Male	1
			Mixed Adult	1
	RNA polymerase II	15	Embryos	8
			Larvae	5
			Pupae	1
			Mixed Adult	1

Detailed information of these datasets can be seen in Additional file 3: Table S2 and Table S5

The next question addressed in this study is whether RNA-seq is a reliable platform for the discovery of novel lncRNAs. A previous study used chromatin immunoprecipitation sequencing (ChIP-seq) data of chromatin signatures to detect active transcription of lncRNAs [15]. Thus, we integrated multiple sets of RNA-seq and ChIP-seq data (Table 1) to investigate transcription of lncRNAs during the development of *D. melanogaster*. We observed that a large proportion of genomic regions for lncRNAs expressed in RNA-seq were not occupied by chromatin signatures (H3K4me3, H3K36me3 and Pol II) that are usually associated with active transcription. However, no studies have discussed which feature (chromatin signatures or expression intensities) is better for inferring the existence of lncRNAs. To answer this question, we designed experiments of quantitative reverse transcriptase-dependent polymerase chain reaction (RT-qPCR) to evaluate the confidence level of lncRNAs discovered from RNA-seq. In summary, this study aims to demonstrate that ambitious integration of sequencing data followed by computational procedures can largely facilitate novel lncRNA discovery as well as enhance lncRNA annotation.

## Results

### Curated lncRNAs in *D. melanogaster*

In this study, a non-redundant set of 1999 lncRNA genes (2347 transcripts) from FlyBase (r5.57) [16] and the UCSC genome browser [17] was first constructed. Next, the long intergenic non-coding RNAs (lincRNAs) reported in the study by Young et al. [18] and Brown et al. [19] were collected to expand the list. Among the 1119 lincRNAs reported by Young et al. and the 3088 lncRNAs by Brown et al., some potentially redundant lincRNAs or lncRNAs were excluded by a selection procedure (see Methods). In the end, 583 lincRNA genes (583 transcripts) from Young et al. and 772 lncRNA genes (1207 transcripts) form Brown et al. were added to the non-redundant set reported in the present study.

**Fig. 1:**
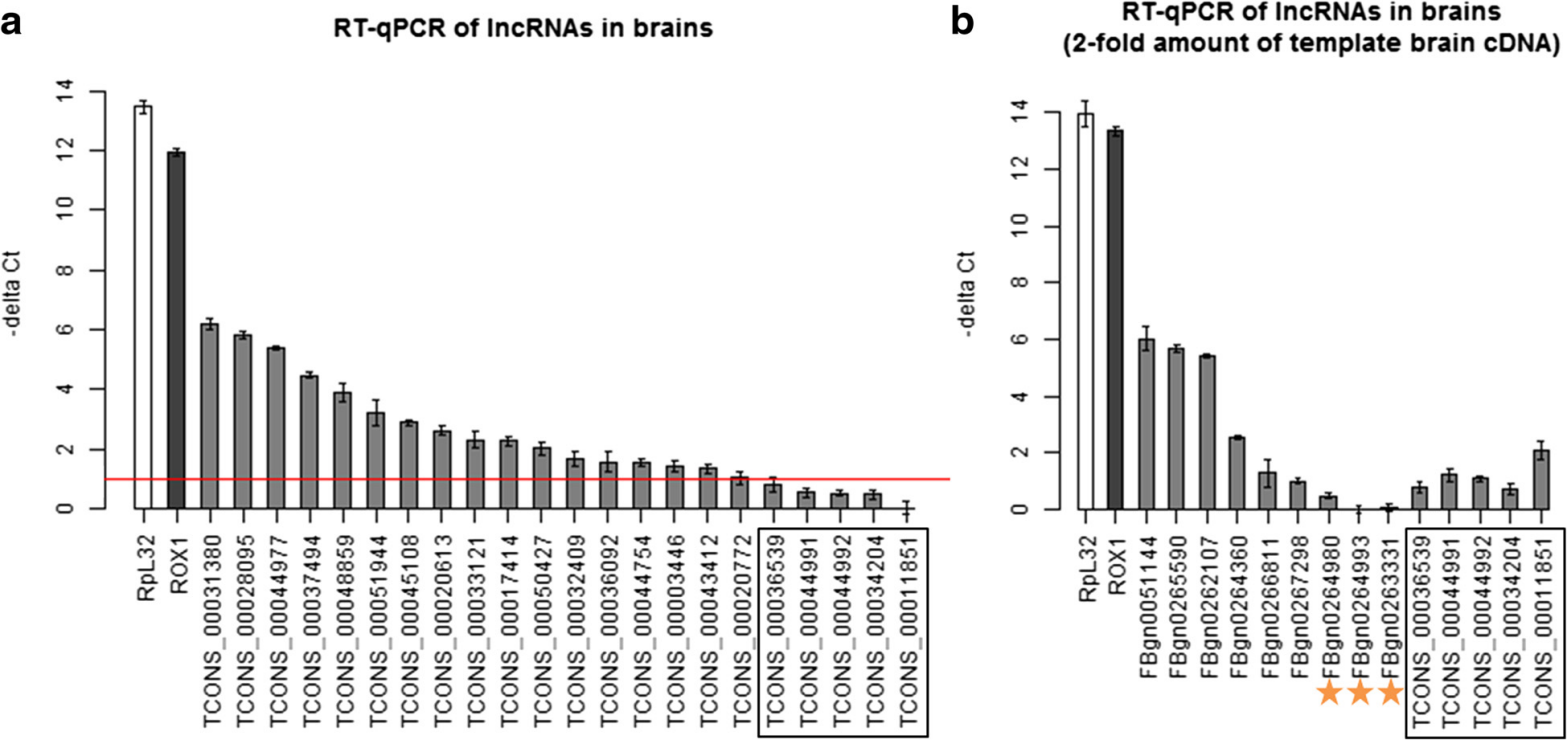
RT-qPCR experiments for a selected set of lncRNAs in brains. **a** 22 novel lncRNAs discovered in the present study were selected for validation. RpL32 (a coding gene) and roX1 (a non-coding gene) were included as positive controls. The horizontal line indicated − delta Ct ≥ 1. The rectangle indicated the five lncRNAs with considerably low expression, and was tested again by the second RT-qPCR experiment shown in (**b**). **b** The five lncRNAs from the rectangle of (**a**) were tested again by RT-qPCR with twofold amount of template cDNA. Ten FlyBase lncRNAs were included for comparison. The three FlyBase lncRNAs highlighted by the orange stars were selected because their RPKM values in our brain RNA-seq data was 0

Additionally, we developed an approach to discover lncRNAs from the brain-specific RNA-seq datasets of fruit fly produced in this study (SRP051132), which were obtained using two types of library construction, the poly(A)-enriched and ribo-zero protocols. The proposed pipeline consists of several steps, including reference-based assembly (using an earlier version of gene annotations downloaded from UCSC genome browser on March 13th, 2013), coding potential estimation, ribosomal RNA exclusion, and read remapping (see Methods). The results consisted of 754 intergenic transcripts that have not been previously annotated. After excluding transcripts with lengths less than 200 bp, 725 transcripts remained as putative lncRNAs. Then, we retained 591 putative lncRNA genes which showed a low potential to encode proteins. After excluding ribosomal RNA contamination, 587 putative lncRNA transcripts remained. We further excluded 57 transcripts that had no sufficient read support during the follow-up read remapping. Before finalizing the list, we compared the discovered lncRNAs with the most updated gene annotations from UCSC genome browser (Sep. 21^st^, 2015), and removed 68 transcripts that overlapped some newly reported coding genes in the sense direction. Finally, we obtained 462 novel lncRNA transcripts that have not been reported previously. To investigate the validity of the discovered lncRNAs, 22 novel lncRNA genes were selected for RT-qPCR experiments applied on fly brains. In Fig. 1(a), the results showed that 17 novel lncRNA genes have adequate expression (−delta Ct ≥ 1). For the five lncRNAs of which the expression was not clear (−delta Ct < 1), we doubled the amount of template brain cDNA and performed RT-qPCR again on these five low-expressed lncRNA genes. In the second RT-qPCR validation experiment, seven FlyBase lncRNA genes that were believed to be expressed in brains and three FlyBase lncRNA genes that were believed to be unexpressed in brains were also included for comparison. The ten FlyBase lncRNAs were selected according to the RPKM values from our poly(A)-enriched RNA-seq data of brain (RPKM > 1 suggested expressed; RPKM = 0 suggested unexpressed). The results in Fig. 1(b) revealed that the expressed and unexpressed FlyBase lncRNA genes showed distinct values in RT-qPCR experiments. When compared with the three unexpressed FlyBase lncRNA genes, the five novel lncRNA genes were also considered expressed in brains.

In total, a set of 3816 curated lncRNA genes (4599 transcripts) in *D. melanogaster* was constructed in this study (Additional files 1 and 2). The average length of the curated lncRNA transcripts is 1008 bp with a diverse range. More than 97 % of the lncRNA transcripts have lengths from 200 bp to 4000 bp (Additional file 3: Table S1).

### Improving the annotation of the lncRNAs reported by Young et al

Young et al*.* [18] reported 1119 lincRNAs for *D. melanogaster* in 2012, but provided no detailed information because the RNA-sequencing reads were not generated with a strand-specific library construction [20]. In this study, we collected the original 30 RNA-seq datasets [20] used by Young et al. (Table 1 and modENCODE IDs: 4433-4462 as shown in Additional file 3: Table S2) and adopted 29 additional stranded poly(A)-enriched RNA-seq datasets at different developmental stages (Table 1 and modENCODE IDs: 4291-4319 as shown in Additional file 3: Table S2) to determine the exon regions and transcriptional directions for the lincRNAs reported in Young et al*.*’s study. After excluding redundant lincRNAs against the annotated lncRNAs from the databases and removed transcripts which are no longer lincRNAs in the current FlyBase annotations (FBrf0220965), 583 lincRNA genes remained. To identify the exon regions of these 583 lincRNA genes, we remapped the 30 RNA-seq datasets to the lincRNA sequences using Cufflinks [21]. We found that most of lincRNA genes from Young et al. consisted of only one or very few exons (Additional file 3: Table S3 and Additional file 4). As for transcriptional direction, similar procedures were conducted. We annotated the direction of transcription in about 67 % of the 583 lincRNA genes from the study by Young et al*.* (Table 2). To be more specific, 200 lincRNA genes were identified on the positive strand and 192 on the negative strand of the fruit fly genome (Table 2 and Additional file 2).

**Table 2:** Statistics of transcriptional direction in the lncRNA genes from different sources. The mRNA information was downloaded from the UCSC genome browser (Sep. 21st, 2015)

Transcriptional direction	FlyBase + UCSC	Young et al.	Brown et al.	Present study	mRNA
Positive (+)	1011	200	392	268	14,941
Negative (-)	988	192	380	194	15,321
Unknown (*)	0	191	0	0	0
Total	1999	583	772	462	30,262

### Utilizing additional RNA-seq datasets to improve the annotation of the 4599 curated lncRNA transcripts

We utilized the RNA-seq datasets from multiple sources as well as those generated in this study to improve the annotation of the curated lncRNAs. Three properties were emphasized here: (1) the classification of a lncRNA in terms of its genome location and transcriptional direction; (2) whether the lncRNA is expressed in the brain or not; and (3) whether the lncRNA has a poly(A) tail or not.

The lncRNAs collected in the present study were classified into several groups according to their genome locations with respect to the closest adjacent coding gene. For lncRNAs located in regions that overlap with coding genes, the transcriptional direction was also considered to be an essential aspect for classification. In this regard, lncRNAs are classified into anti-sense exonic, sense exonic, anti-sense intronic and sense intronic lncRNAs, according to the transcriptional direction with respect to the overlapping coding gene. Among the curated 4599 lncRNA transcripts, 2602 were classified as intergenic lncRNA transcripts, 1100 as exonic lncRNA transcripts (Table 3 and Additional file 2) and 706 as intronic lncRNA transcripts. There were 191 lncRNA transcripts for which the transcriptional direction could not be determined and were classified as ‘unknown’. Table 3 shows that the number of lncRNAs for the four groups decreased as follows: anti-sense exonic lncRNAs > anti-sense intronic lncRNAs > sense exonic lncRNAs > sense intronic lncRNAs. The lncRNA numbers of the four groups in the different euchromatin regions were also provided (Additional file 3: Figure S1). Here, we only considered lncRNAs located in euchromatin because most lncRNAs were expressed from the euchromatin in fruit fly.

**Table 3:** Types of lncRNA transcripts

Types	Number of lncRNAs	Averaged length (±sd)	Number of exons (counts of lncRNAs)	Transcriptional direction (counts of lncRNAs)
Intergenic	2602	1002 (±1305.81)	Single (1805); multiple (797)	+(1375); −(1227)
Exonic				
Anti-sense	832	1161 (±1059.20)	single (373); multiple (459)	+(448); −(384)
Sense	268	1380 (±1317.87)	single (154); multiple (114)	+(131); −(137)
Total	1100			
Intronic				
Anti-sense	495	770 (±581.83)	single (292); multiple (203)	+(239); −(256)
Sense	211	733 (±633.81)	single (149); multiple (62)	+(108); −(103)
Total	706			
Unknown	191	813 (±782.66)	Single (164); multiple (27)	NA
Total	4599			

+: positive strand

−: negative strand

*NA* not available

Additionally, this study provided two sets of sequencing reads of RNA samples from the brain (Table 1). With the two datasets, we could infer which lncRNAs were expressed in the brain. If the criterion ‘RPKM > 1’ was used, the data revealed that about one third of lncRNAs (1464 transcripts, Additional file 2) were expressed in the brain. In Fig. 1(b) we showed the RT-qPCR experiments of seven lncRNA genes with RPKM > 1 and three lncRNA genes with RPKM = 0. The RT-qPCR results showed that the − delta Ct values of the seven lncRNA genes with ‘RPKM > 1’ were distinguishable from the three lncRNA genes with ‘RPKM = 0’. In this regard, ‘RPKM > 1’ is considered as a safe criterion to infer the expression of lncRNAs in the brain. Next, we further examined whether a lncRNA contains the poly(A) tail. Both poly(A)-enriched and ribo-zero library constructions were used in the present study because some lncRNAs were previously found to contain no poly(A) tails in mammals [22–24]. Among the 1464 lncRNA transcripts observed in the brain RNA-seq data, there were 190 lncRNA transcripts with a high probability of not containing poly(A) tails when expressed in the brain (Additional file 2).

### Supporting evidence for the collected and the newly discovered lncRNAs

Existing data of chromatin signatures and expression profiles of *D. melanogaster* were applied to examine the associated chromatin modifications and the expression levels of lncRNAs. For each lncRNA, the presence of transcription-related chromatin signatures was provided in Additional file 2.

#### Expression profiles

**Fig. 2:**
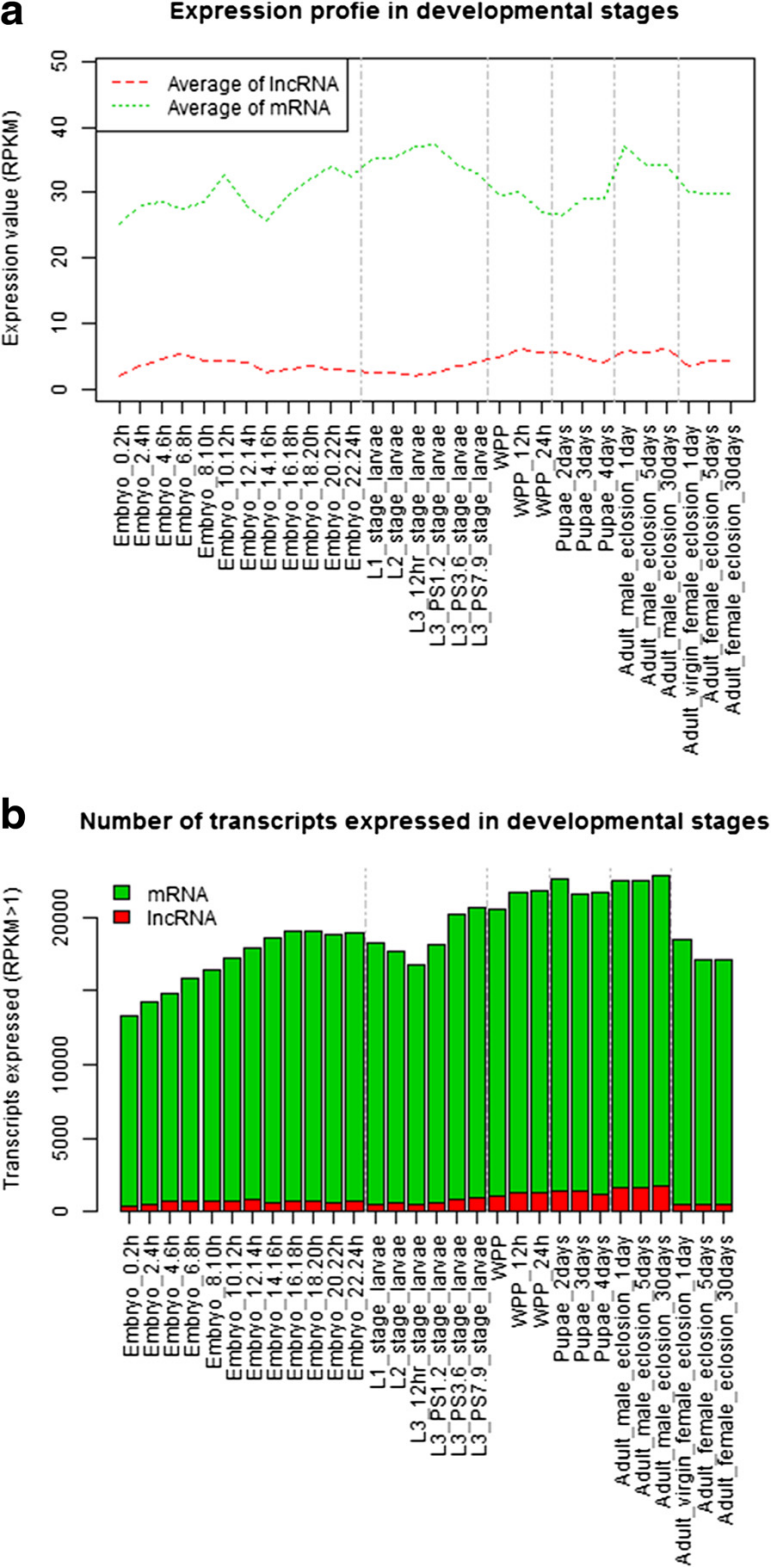
Expression profiles at different developmental stages of fruit fly. **a** Averaged RPKM values at different developmental stages for lncRNAs and mRNAs. **b** Numbers of expressed transcripts (RPKM > 1) at different developmental stages for lncRNAs and mRNAs, respectively

To quantify the expression level of lncRNAs, the RPKM value of every lncRNA transcript at each developmental stage was calculated along with the averaged values of all lncRNA molecules and the averaged values of all mRNA molecules. Figure 2(a) shows that mRNA, on average, had ~8-fold higher expression than lncRNA at each developmental stage. Moreover, Fig. 2(b) shows that the numbers of transcripts expressed at the developmental stages are similar to those reported in the original study [20]. On average, lncRNA molecules occupied ~4.3 % of all transcripts expressed at the developmental stages.

#### Chromatin signatures

**Fig. 3:**
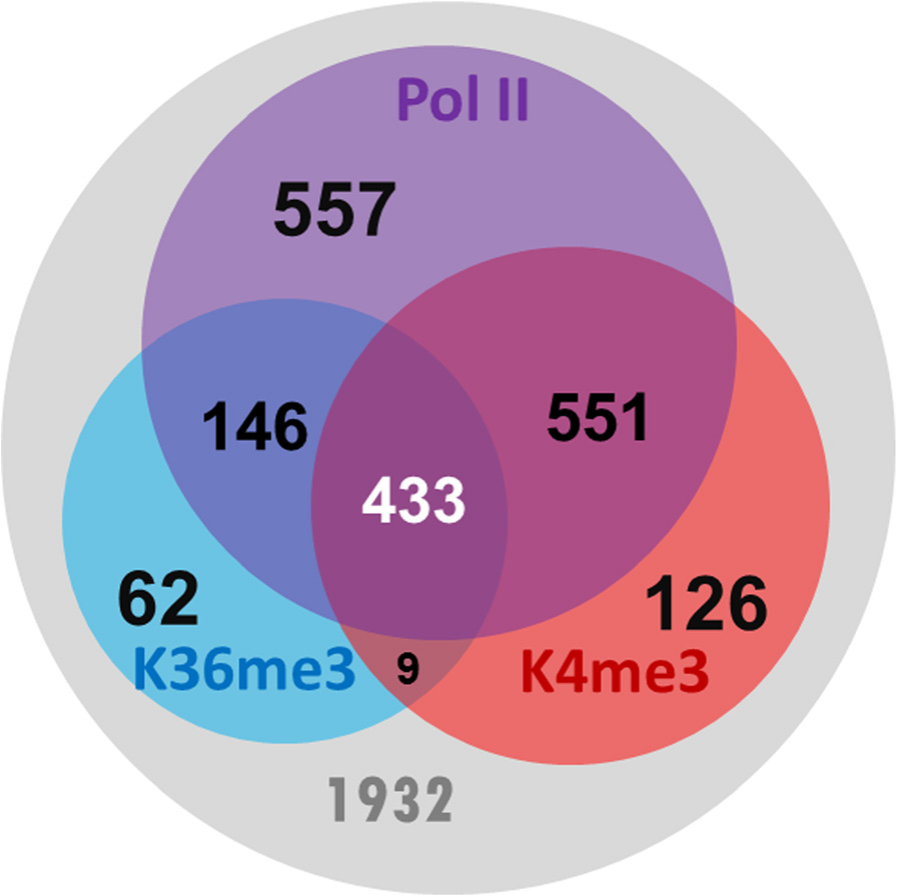
Analysis of chromatin signatures (Pol II, H3K36me3 and H3K4me3) in the curated lncRNA genes

In the set of curated lncRNAs, 1119 of the 3625 lncRNA genes with well-defined transcriptional direction had a detectable H3K4me3 signal at the proximal region of the genes (Fig. 3). In addition, 650 lncRNA genes had detectable H3K36me3 signals, covering, on average, ~70 % of the transcribed regions. We also examined the Pol II ChIP-seq data and found that 1687 (44 %) lncRNA genes had Pol II signals with an average coverage of ~60 % over the transcribed regions. In summary, 433 lncRNA genes showed ‘K4–K36’ and Pol II signatures, strongly suggesting that these lncRNAs were epigenetically regulated like protein coding genes. We were aware of the possibility that the chromatin signatures assigned to the lncRNA genes were actually associated with the overlapped coding genes. There are 340 sense exonic/intronic lncRNA genes that may encounter such a situation.

In combination with the information of expression profiles and chromatin signatures, we found that a large proportion of expressed lncRNA transcripts (RPKM > 1) were not occupied by H3K4me3, H3K36me3 and Pol II chromatin signatures, which are believed to be present in the actively transcribed regions. The inconsistency between chromatin signatures and RNA-seq expression raises the question as to whether the identified lncRNAs were really transcribed or not. We addressed this issue in the following paragraph.

### Experimental validation of a selected set of lncRNAs by RT-qPCR

**Fig. 4:**
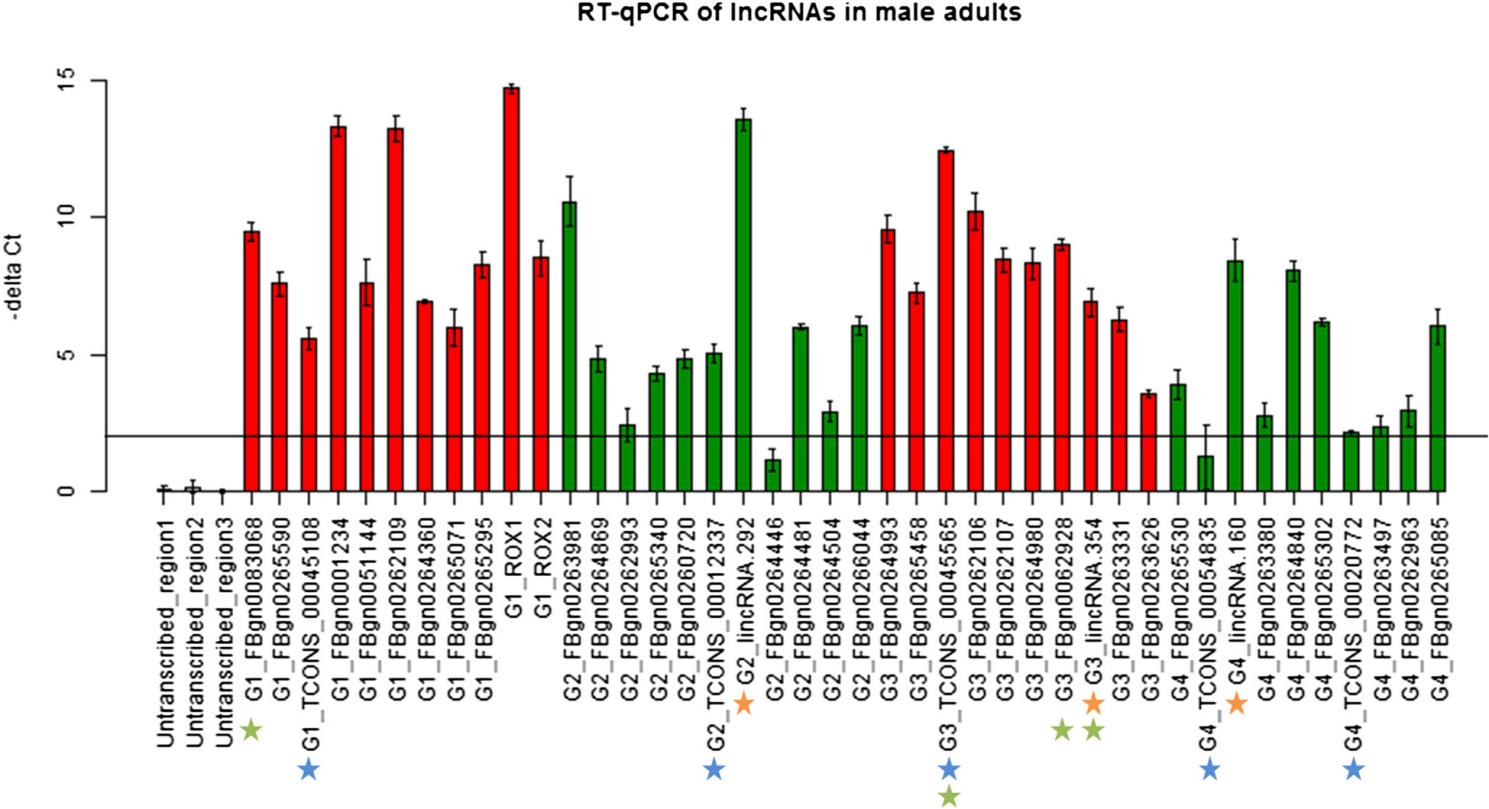
RT-qPCR experiments of a selected set of lncRNAs in male adults. G1: high expression with chromatin signatures (11 lncRNAs); G2: low expression with chromatin signatures (11 lncRNAs); G3: high expression without chromatin signatures (10 lncRNAs); and G4: low expression without chromatin signatures (10 lncRNAs). Three negative controls (un-transcribed region 1, 2, and 3) were all around zero. Stars were used to highlight the lncRNAs that were not from the databases (Orange stars: the selected lncRNAs from Young et al. [18]. Blue stars: the lncRNAs from the present study). The horizontal line indicated the cutoff (−delta Ct ≥2) used to define a validated lncRNA. Green stars: the transcripts that are now annotated as other types of transcripts by FlyBase, and thus were removed from the list of the curated lncRNAs in the present study

To investigate whether the collected lncRNA genes were indeed actively transcribed, we used RT-qPCR to detect the expression of a selected set of lncRNAs in adult male flies. A set of lncRNAs expressed in adult male flies (RPKM >1) were selected and divided into four groups according to two properties: (a) lncRNAs with all of the three chromatin signatures (H3K4me3, H3K36me3 and Pol II) or without any of the three chromatin signatures, and (b) lncRNAs with high expression (RPKM > 3rd quartile, i.e., 12.92) or with low expression (RPKM < 1st quartile, i.e., 2.78). In each group, at least 10 lncRNAs were randomly selected to be validated with RT-qPCR. The four groups were defined as (G1) high expression with chromatin signatures (11 lncRNA genes), (G2) low expression with chromatin signatures (11 lncRNA genes), (G3) high expression without chromatin signatures (10 lncRNA genes) and (G4) low expression without chromatin signatures (10 lncRNA genes). Surprisingly, the transcripts of almost all lncRNA genes (95.24 % of all tested lncRNA genes) were detectable except for one lncRNA gene in G2 and one lncRNA gene in G4 (Fig. 4 and Additional file 3: Table S4). Among the validated lncRNA genes, three lincRNA genes (lincRNA.354 is now annotated as a protein-coding gene in FlyBase) were discovered by Young et al*.* [18] and five lncRNA genes (TCONS_00045565 is now annotated as an rRNA gene in FlyBase) were reported by the present study. The RT-qPCR results confirmed that most of the lncRNA genes identified by RNA-seq are not transcriptional noise. Furthermore, our results suggested that the lack of associated H3K4me3, H3K36me3 and Pol II signatures might not directly imply no active transcription of lncRNAs, since most of the expressed lncRNA genes without these three chromatin signatures (G3 and G4) were successfully detected by RT-qPCR.

## Discussion

In this study, we compiled an up-to-date list of fruit fly lncRNAs from databases and literature and found that the number of known lncRNA genes in fruit fly (~3300) was much smaller than those reported in human (~56,000) and mouse (~46,000) [25]. We suspected that the set of known lncRNAs in fruit fly was far from exhaustive. Indeed, 462 novel lncRNA genes were discovered when two brain-specific RNA-seq datasets were produced in the present study. Thus, more lncRNA genes will likely be found when more RNA-seq studies of fruit fly are conducted in the future.

In order to discover lncRNAs that do not contain poly(A) tails, we have developed a computational approach to identify novel lncRNAs by integrating sequencing read datasets from two different library construction protocols, the poly(A)-enriched and ribo-zero protocols. This approach can be applied to future studies for the same purpose. The final set of curated fly lncRNAs contain 3816 lncRNA genes (4599 lncRNA transcripts), which is larger than the 2460 lncRNA genes in FlyBase (Release 6.06 [16]), and the 2446 lncRNA transcripts recently reported by Matthews et al. [26]. Our final list is also larger than the latest version (version 4) of a well-known lncRNA database, NonCode (961 lncRNA genes) [25]. The present study also demonstrated that novel lncRNAs can be found in a tissue-specific manner, as suggested by a previous study in mammals [14]. We found that 33 % of the 3816 lncRNA genes were expressed in the brain, when the criterion ‘RPKM > 1’ was used. This number is considerably higher than that observed in other tissues reported by Brown et al*.* [19]. The study of Brown et al*.* incorporated RNA-seq data from 10 types of tissues and the testis tissue showed the highest number of expressed lncRNA genes (~30 % of the 1875 lncRNA genes).

To investigate the quality of the lincRNAs discovered in the present study, we conducted three analyses and selected a set of lincRNA genes for RT-qPCR validation to investigate the reliability of these newly discovered lncRNAs. For a lncRNA, it was examined whether (1) it was observed to be expressed in the collected RNA-seq datasets from developmental stages; (2) it was predicted with a low coding probability by another predictor; and (3) it was not predicted to contain any conserved domains of proteins. As shown in Additional file 5, 86.15 % of the 462 novel lncRNA genes discovered from fly brain were also observed expressed in at least three developmental stages. In the proposed workflow of discovering lncRNAs, we applied a SVM-based prediction tool, Coding Potential Calculator (CPC) [27], to filter out potential coding sequences. Here, we applied another tool for estimating coding potential, Coding-Potential Assessment Tool (CPAT) [28], on the discovered lncRNAs. The result (Additional file 5) showed that only seven transcripts were with a coding probability ≥ 0.39. This cutoff threshold 0.39 was an optimum cutoff for fruit fly suggested by Wang et al. [28], where 96 % of fly coding genes were shown to have a coding probability ≥ 0.39 (data shown on the tool download page). Moreover, the results of invoking RPS-BLAST showed that only nine newly discovered lncRNA transcripts might contain conserved domains from the Conserved Domains database (CDD, version 3.4), as shown in Additional file 5 as well. Finally, the RT-qPCR validation for the selected novel lincRNA genes suggested that all of the 22 novel lincRNA genes were shown to be expressed in brains when compared with the negative controls (Fig. 1). This reveals the reliability of the discovered novel lncRNA genes.

In the curated list, we observed that there are some lncRNA transcripts from different sources partially sharing common genomic regions. These lncRNA transcripts might be in fact the same lncRNA, might be different splicing forms of a single lncRNA gene, or might be actually independent lncRNA genes. We realized that it remained difficult to learn the fact and determine the exact boundaries for these putative lncRNAs based on the limited information collected so far. Before a mature methodology can be developed, manual examination on RNA-seq data in a genome browser is highly recommended. We highlighted the overlap information in Additional file 2 to remind the readers that more investigations on such lncRNAs are needed. In addition, we also observed that the types of lncRNA transcripts (exonic, intronic, or intergenic lncRNAs) would potentially be changed once the annotation of protein-coding genes is updated. As the loci and boundaries of protein-coding genes continue to be refined, noncoding RNAs originally classified as intergenic may be found to be exonic, intronic or even become a new splicing form of a coding gene. Some of the Young et al. lincRNAs have been found by a follow-up FlyBase analysis (FBrf0220965) to overlap UTRs and are probably not lncRNAs. Therefore, the readers should be aware that the number of exonic sense lncRNAs in the curated list might be inflated by these lncRNAs.

**Fig. 5:**
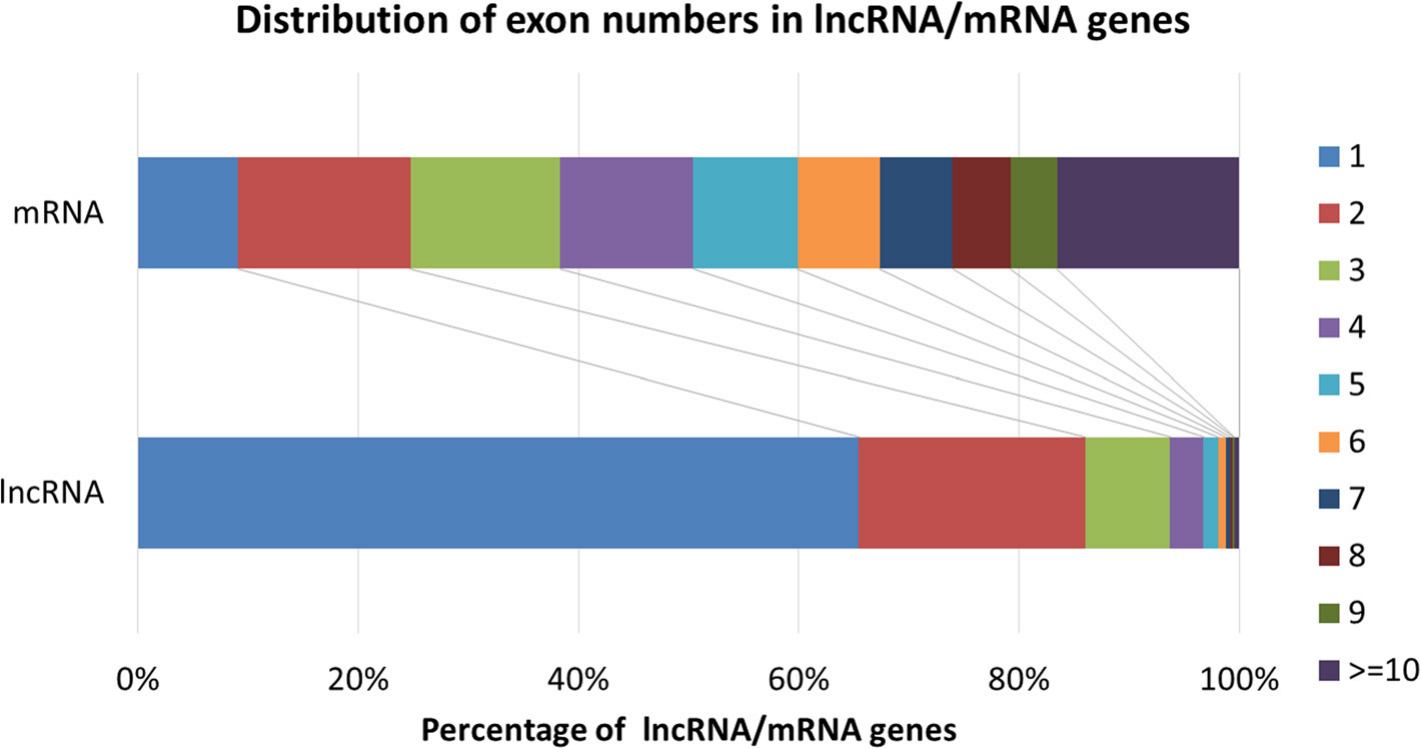
Distribution of exon numbers in the lncRNA/mRNA genes

This study used additional RNA-seq data from the modENCODE database to improve the annotation regarding transcriptional direction (Table 2 and Additional file 2) and the number of exons (Additional file 3: Table S3 and Additional file 4). When comparing lncRNAs with fruit fly mRNAs, we found that about half of the curated lncRNA genes were transcribed in the positive strands and half in the negative strands (Table 2). For each specific group of the lncRNA transcripts in Table 3, the lncRNA transcripts were equally derived from both strands. Moreover, 988 lncRNA genes (25.89 % among the 3816 lncRNA genes) were found to be transcribed in a direction antisense to protein coding genes. This number is larger than that (15 %) reported in human [29]. Again, by the follow-up FlyBase analysis (FBrf0220965), some of the Young et al. lincRNAs have been found to actually consist of two or more independent lncRNA genes which map to opposite strands. We observed that the characterization process performed in the present study failed to clarify these cases based on the stranded RNA-seq data collected so far. In this regard, the readers should be aware that such complicated cases were not easily to be discovered automatically by the proposed computational approach, and might be still present in the remaining 583 Young et al. lincRNA genes curated in the list. As for the number of exons in lncRNAs, fruit fly lncRNAs tend to have fewer exons than mRNAs (Additional file 3: Table S3), which is consistent with the observation in rat by Wang et al. [30]. Figure 5 shows that ~60 % of mRNAs contain no more than five exons. The percentage of mRNAs with different exon numbers were roughly equally distributed (9 % for one exon, 16 % for two exons, 14 % for three exons, 12 % for four exons and 9 % for five exons). In contrast, ~94 % of lncRNAs contain one to three exons, and more than half of the lncRNAs contain only single exon. The exon numbers of lncRNAs were apparently smaller than that of mRNAs. It is not clear whether this was because the average length of the curated lncRNAs (1008 bp) is shorter than that of mRNAs (2869 bp). Additionally, in Table 3, we showed that intergenic lncRNAs were the major type of lncRNAs that contained only one exon.

Next, we utilized the peak detection results of 34 CAGE datasets from the study of Brown et al*.* to investigate the 5’ end completeness of the curated lncRNA transcripts. The result showed that about ~55 % of the curated lncRNA transcripts can find a CAGE peak within the ±50-bps region with respect to the 5’ end of lncRNA transcripts (Additional file 2). Generally speaking, our study shows that integrating multiple public datasets provides useful information for fly lncRNAs.

In the present study, the influence of RNA-seq data with two different types of library constructions, poly(A)-enriched and ribo-zero libraries, was also investigated. The data showed that 190 lncRNA transcripts were only detected in the reads from the ribo-zero library, but not in the reads from the poly(A)-enriched library. This indicates that some lncRNA transcripts do not contain poly(A) tails when they are expressed in the brain. Such lncRNA transcripts can be detected only by the ribo-zero library construction.

Moreover, to study whether the lncRNAs reported by RNA-seq were associated with chromatin modifications, we collected ChIP-seq datasets of the three chromatin signatures, H3k36me3, H3k4me3 and Pol II, which are known to be strongly associated with active transcription [4–6]. The collected datasets involved samples from embryos, larvae, pupae and adults of *D. melanogaster*, with the exception of H3k36me3 datasets in which pupae were not found. Furthermore, RNA-seq datasets of fly samples at different developmental stages were included to quantify the expression of lncRNAs. We found that a large proportion of the expressed lncRNAs (RPKM >1) were not occupied by chromatin signatures (H3K4me3, H3K36me3 and Pol II). This observation raised the question of whether RNA-seq is a reliable platform for detecting transcription of lncRNAs, because false detection of lncRNAs might happen due to contaminated genomic DNA during library construction. As both the inference of lncRNA expression and signatures of active transcription were obtained using high-throughput technologies, we used RT-qPCR to detect the transcription of lncRNAs.

In the RT-qPCR experiments, we selected 42 lncRNA genes reported by RNA-seq. The results revealed that most lncRNA genes (95.24 %) were indeed present at the chosen stage (male adults) of the fruit flies (Fig. 4). Two known lncRNA genes expressed in male adults, roX1 and roX2 [10], were also validated by RT-qPCR. These observations provided strong support that RNA-seq is a reliable tool to identify lncRNA genes. In addition, we divided the 42 selected lncRNA genes into four groups with all possible combinatorial conditions of chromatin signatures (present or absent) and expression (high or low). The data showed that in all four groups, all lncRNA genes except two with low expression could be successfully detected by RT-qPCR. This observation held even for the expressed lncRNA genes that had none of the three chromatin signatures. However, it should be noted that the collected ChIP-seq datasets were not sampled from the stages as precisely as the RNA-seq datasets, which were collected from 30 time points (12 for embryos, 6 for larva, 3 for white pupae, 3 for pupae, 3 for male adults and 3 for female adult stages) during the development of *D. melanogaster*. The inconsistency between RNA-seq and ChIP-seq data may be because the collected ChIP-seq data were not extensive. In particular, ChIP data of H3K36me3 sampled from pupae was not found during data collection.

## Conclusions

In this study, we have developed a procedure to discover novel lncRNAs using RNA-seq technology, and used a large number of RNA-seq datasets as well as lncRNA databases and ChIP-seq datasets to improve the annotation of lncRNAs in fruit fly. From these efforts, we have provided an enlarged set of *D. melanogaster* lncRNAs, including known lncRNAs and novel lncRNAs from the two tissue-specific RNA-seq datasets generated in this study. The novel lncRNAs we identified suggests that many fruit fly lncRNAs remain to be identified. Moreover, we have also improved the annotation of the curated lncRNAs regarding transcriptional direction, exon regions, classification, expression in the brain, possession of a poly(A) tail, and presence of conventional chromatin signatures by utilizing the strand-specific RNA-seq and the ChIP-seq datasets from the modENCODE database and data from the present study. Through RT-qPCR experiments, we demonstrate that RNA-seq is a reliable platform to discover lncRNAs. In summary, the present study provided a firm foundation for studying the functions of lncRNAs in *Drosophila*.

With the improved annotation of transcriptional direction, researchers can possibly retrieve the promoter regions of lncRNAs and investigate the potential regulators that regulate lncRNA expression. Moreover, this information can be used to investigate the co-expression relationships between lncRNAs and coding genes in order to further understand the functional roles of the set of curated lncRNAs. In conclusion, the present study has integrated many RNA-seq and ChIP-seq datasets to increase the compilation breadth and annotation detail of lncRNAs. The set of curated lncRNAs along with improved annotation can serve as an important resource in lncRNA studies.

## Methods

### Collection of published lncRNAs

The lncRNAs were collected from FlyBase [16], the UCSC genome browser [17], Young et al*.* [18], and Brown et al. [19]. A set of lncRNAs was obtained using the keyword term “non_protein_coding_genes” when querying FlyBase *D. melanogaster* (r5.57). LncRNA transcripts shorter than 200 bp were filtered out. First, the lncRNA transcripts from FlyBase were chosen as the primary set of lncRNA sequences. Second, BLASTn [31] was used to align the lncRNA transcripts collected from the UCSC genome browser against the primary set. Afterwards, by checking the alignments with E-value < 10^-10^ in the BLASTn results, redundant lncRNA transcripts were removed when either of the following two conditions was satisfied: (1) a lncRNA has the same loci with another lncRNA, or (2) a lncRNA overlaps another lncRNA with an overlapping region covering 50 % of the transcript length. With the specified criteria, 972 redundant sequences were excluded. Third, 1119 lincRNAs were collected from the study by Young et al*.* [18], where 415 sequences were excluded because they contained overlapping regions with the non-redundant set of lncRNA transcripts from FlyBase and the UCSC genome browser. Additionally, 3088 lncRNA transcripts were collected from Supplementary Data 2 of the study of Brown et al*.* [19]. We removed 49 lncRNA transcripts with a length < 200 bp and 19 transcripts that were annotated as coding genes in the file provided by Brown et al. The remaining 3020 lncRNA transcripts were next aligned to the above non-redundant set of lncRNA transcripts from FlyBase, UCSC, and Young et al. by using BLASTn. The alignments with E-value < 10^-10^ in the BLASTn results were further examined by the following selection procedure. We removed lncRNA transcripts that were annotated with an already included FlyBase lncRNA ID. LncRNA transcripts containing overlapping regions with the curated FlyBase/UCSC lncRNA transcripts (covering >50 % of the either transcript length) were removed unless the new lncRNA transcripts contain multiple exons and the number of exons differs from that of FlyBase/UCSC lncRNA transcripts. Afterwards, lncRNA transcripts aligned to lncRNA transcripts of Young et al. were removed only if they have the same loci or have an overlapping region covering 90 % of transcript length. As a result, 1635 redundant lncRNA transcripts were removed. All lncRNA transcripts were then aligned to 156 ribosomal RNAs collected from FlyBase r6.07 (2 sequences) and the NCBI database (154 sequences) using BLASTn. Sequences (10 sequences) with E-value < 10^-10^ and identity > 99 % were removed to exclude ribosomal RNA contamination.

To ensure that the lncRNAs curated in this study did not contain newly reported coding genes present in the most updated FlyBase annotations, we retrieved ‘Feature Type’ and ‘Gene Model Status’ for the curated lncRNA transcripts from FlyBase by submitting transcript IDs to the batch download tool of FlyBase r6.07. Additionally, we utilized ‘Coordinates Converter’ provided by FlyBase to see whether a transcript location is no longer present in the release 6 genome (R6). Moreover, for the lncRNA transcripts from Young et al., FlyBase recently incorporated these lncRNA transcripts and provided update annotations based on a manual review (FBrf0220965). By taking the above-mentioned information from FlyBase into account, we removed 673 transcripts that were annotated as protein coding genes, pseudogenes, rRNA genes, snRNA, snoRNA, scaRNA, out-of-date IDs, or located within TE regions or the sequences dropped by the BDGP in the R6 genome. In the end, this study constructed a set of lncRNAs from FlyBase, the UCSC genome browser, and the studies by Young et al*.* [18] and Brown et al. [19], consisting of 3354 lncRNA genes, corresponding to 4137 lncRNA transcripts.

### RNA-seq data of the fly brain

Brain samples were collected from 4-day post-eclosion *Canton S* male adults. At a time, 20 to 30 adults were gassed with carbon dioxide and dissected. The collected brains were preserved in refrigerator until 100 brains were collected. Afterwards, total RNA was purified from the 100 brains, using the NucleoSpin® RNA II Purification Kit. RNA-seq was performed using the strand-specific library with poly(A)-enriched protocol or Ribo-Zero™ Gold Kit to generate paired-end 90-bp reads on the Illumina Hi-seq 2000 platform. In total, ~25 million and ~50 million pair-end reads of 90-bp in length were obtained from the poly(A)-enriched library and the total RNA (with Ribo-Zero™ Gold Kit) library, respectively. The raw reads have been submitted to NCBI Sequence Read Archive database (SRP051132).

### Novel lncRNA discovery

**Fig. 6:**
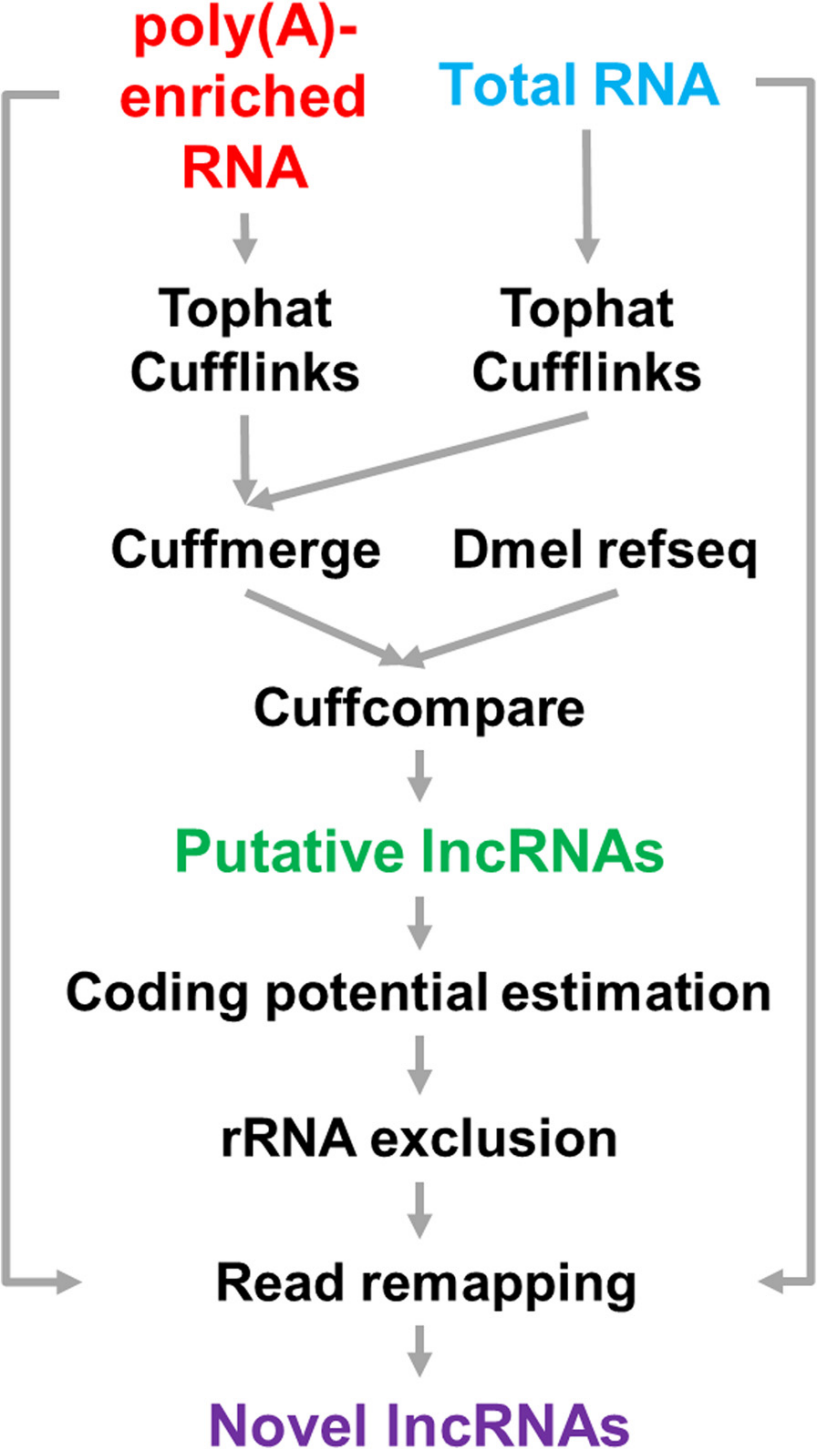
Procedures for discovering novel lncRNAs from RNA-seq data of the present study. The sequencing read datasets of poly(A)-enriched RNA and total RNA were respectively mapped to the reference genome sequence using TopHat and Cufflinks. Putative lncRNAs were then discovered by Cuffcompare, followed by coding potential estimation and rRNA exclusion. Sequencing reads were again mapped to the set of putative lncRNAs to construct the final set of novel lncRNAs

To discover novel lncRNAs from the two new datasets described above, we first mapped all short reads onto the unmasked *D. melanogaster* genome sequences (BDGP R5/dm3; from the UCSC genome browser), using TopHat [21]. Cufflinks [21] was then used to assemble the mapped reads and the assembled transcripts were compared to the reference annotation (Dmel refseq) from the UCSC genome browser (downloaded on March 13th, 2013) using Cuffcompare, a utility included in Cufflinks. The two sets of assembled transcripts, from poly(A)-enriched RNA and total RNA, respectively, were compared to the reference annotation at the same time to get a union set of intergenic transcripts. We set a length of 200 bp as the cutoff to exclude shorter non-coding RNAs. We then calculated the coding potential of all putative lncRNA loci using the Coding Potential Calculator (CPC) [27]. The putative lncRNA transcripts were then aligned against a set of ribosomal RNAs (the same set described in the “Collection of published lncRNAs” section) to exclude ribosomal RNA contamination. Afterwards, we remapped both poly(A)-enriched RNA and total RNA sequencing reads to the putative lncRNA transcripts, using Cufflinks. After remapping, we excluded transcripts with no read support as reported by Cufflinks. The developed computational pipeline is shown in Fig. 6. Then, we compared the identified lncRNAs with the most updated R5 genome annotations downloaded from the UCSC genome browser (Sep. 21^st^, 2015), and removed lncRNA transcripts that overlapped with some newly reported coding genes in a sense direction. The resulting set of putative lncRNA transcripts were then compared to the set of non-redundant lncRNA transcripts collected from FlyBase, the UCSC genome browser, and the studies by Young et al. [18] and Brown et al. [19] to remove redundant sequences.

### Improving the annotation of curated lncRNAs

To understand the characteristics of the collected and the newly discovered lncRNAs, we integrated information on transcriptional direction, exon regions, classification, expression in the brain, and possession of a poly(A) tail as follows.

#### Transcriptional direction and exon regions

We determined the transcriptional direction and exon regions of each lncRNA based on the existing annotation from databases as well as the strand-specific RNA sequencing data, from both the present study and the modENCODE database [32]. For the lncRNAs discovered in the present study, both sequencing reads from poly(A)-enriched and total RNA libraries were generated by a strand-specific protocol, so that the transcriptional direction and the exon regions of the assembled transcripts could be determined by Cufflinks. As for the lincRNAs from the study by Young et al*.* [18], 29 stranded poly(A)-enriched RNA-seq datasets sampled from different developmental stages and multiple tissues (modENCODE IDs: 4291-4319 as shown in Table 1 and Additional file 3: Table S2) were additionally collected and used to determine the transcriptional direction and exon regions, as the RNA library construction of the datasets originally used by Young et al*.* [18] was not strand-specific.

#### Classification of lncRNAs

**Fig. 7:**
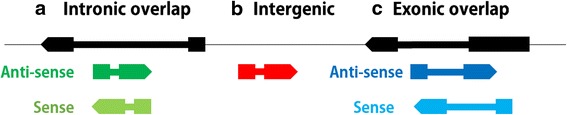
Rules for classifying lncRNAs. Black arrows (transcripts) represent coding genes and colored transcripts are lncRNAs. **a** lncRNAs with intronic overlaps. This group includes lncRNAs (*dark green and light green transcripts*) located in intronic regions of coding genes (*black transcripts*). **b** Intergenic lncRNAs. This group includes lncRNAs (*red transcripts*) located in regions between two coding genes (*black transcripts*). **c** lncRNAs with exonic overlaps. This group includes lncRNAs (*dark blue and light blue transcripts*) overlapping exonic regions of coding genes (*the black transcript*)

Based on the relative location and direction to the closest adjacent coding gene, we divided the lncRNA transcripts into three major classes by in-house perl scripts: (a) lncRNAs imbedded in the introns of protein-coding genes are classified as intronic lncRNAs; (b) lncRNAs that do not overlap with any coding genes are classified as intergenic lncRNAs; and (c) lncRNAs that overlap with an exon in protein-coding genes are classified as exonic overlapping lncRNAs (Fig. 7). All exonic and intronic overlapping lncRNAs were then subdivided into sense and antisense depending on the direction of the protein-coding gene. Unclassified lncRNAs were denoted as an unknown group. Here, as in Young et al*.* [18], we used the annotated gene reference from the UCSC genome browser (Sep. 21^st^, 2015).

#### Expression in the brain

As the sequencing reads of the present study were sampled from the brains of fruit flies, we could thus tell whether a lncRNA was expressed in the brain or not. For each of the sequencing read datasets produced in the present study, the two paired-end sequencing reads (read 1 and read 2) were first concatenated into one read set. Next, we remapped the reads onto the transcript set of the collected and the newly discovered lncRNA transcripts using Bowtie [33] followed by eXpress [34] to normalize the read counts of transcripts as Reads Per Kilobase of transcript per Million mapped reads (RPKM). The lncRNA transcripts with a RPKM greater than 1 were defined as “expressed”.

#### Possession of a poly(A) tail

To answer the question regarding whether a poly(A) tail is required for an expressed lncRNA, the sequencing reads of the present study were generated by using two types of library construction: one was enriched by poly(A) tails (poly(A)-enriched protocol), while the other (ribo-zero protocol) was not. These two types of sequencing reads were quantified with the same procedure as described in ‘Expression in the brain.’ Then, we adopted a stringent criterion to define the group of expressed lncRNA transcripts containing no poly(A) tail if they were expressed in the ribo-zero RNAs (RPKM > 1) but not in the poly(A)-enriched RNAs (RPKM = 0). A stringent criterion is adopted because total RNA sequencing reads with ribo-zero library construction may include mature mRNAs (the major group of RNAs containing poly(A) tails), immature RNAs, partially transcribed RNAs, small RNAs, lncRNAs, etc.

### LncRNA expression during development of *D. Melanogaster*

The gene expression profile of each lncRNA was measured by Illumina sequencing reads of 30 developmental stages (modENCODE IDs: 4433-4462 as shown in Additional file 3: Table S2), from 0-2 h embryos through 30-day male and female adults, provided by Graveley et al*.* [20]. The sequencing reads were pre-processed by trimming 10 bp from the 5’ end to eliminate random primer effects [35]. Bases from the 3’ end were also trimmed until a quality score higher than 20 was reached. In addition, only reads that were at least 36 bp in length were retained for subsequent analysis. The qualified reads were then mapped onto all transcripts including both mRNA and lncRNA sequences using Bowtie [33] and the read counts of transcripts were normalized as RPKM using eXpress [34].

### Chromatin signatures for the expressed lncRNAs during development of *D. Melanogaster*

Like protein coding mRNAs, many expressed lncRNAs in mammalian cells contain a ‘K4–K36’ signature [36]. That is, H3K4me3 is present in the promoter region, followed by a longer stretch of H3K36me3 extending throughout the entire transcribed region. In this study, we integrated the ChIP-seq data containing information of ‘K4–K36’ histone modifications to further characterize the collected lncRNAs. To assign H3K4me3 signals to an lncRNA, we defined regions 500 bp upstream and 100 bp downstream, with respect to the transcription start site (TSS), as the promoter region and used pre-defined protein binding sites from H3K4me3 ChIP-seq datasets collected from modENCODE [32]. Next, we examined H3K36me3 modifications and calculated the coverage as a percentage of the transcribed region in a lncRNA that was covered by the H3K36me3 signal. In addition, as Pol II occupancy can also reveal expression of transcripts, we also considered Pol II occupancy across the promoter region and the transcribed region for a lncRNA as an essential chromatin signature. The modENCODE IDs of all ChIP-seq datasets used in this study are listed in Additional file 3: Table S5. The specific definition of occupied regions for each chromatin signatures is shown in Additional file 3: Figure S2.

### Experimental validation by RT-qPCR

In this study, real-time quantitative PCR (RT-qPCR) experiments were adopted for validating the expression of two selected lncRNA sets in two types of samples, brains and whole bodies of young male adults (*Canton S*). Total RNA samples were purified from 100 brains and 20 whole bodies, respectively, by using TRIzol (Invitrogen) and were subsequently treated with DNase to eliminate genomic DNA contamination. Next, 1 μg of total RNA were converted to cDNA by random hexamer primers and SuperScript™ reverse transcriptase (Invitrogen) according to manufacturer’s protocol along with a negative control without reverse transcriptase. A primer pair for each of the selected lncRNAs was designed, using the Primer-BLAST tool provided by NCBI [37]. The functionality of the designed primer pairs was pre-tested by polymerase chain reactions applied on the genomic DNA purified from 5 *Canton S* larvae. The tests revealed that 35 primer pairs (used in Fig. 1) and 42 primer pairs (used in Fig. 4) worked well which were then used in subsequent analysis (the primer list is shown in Additional file 3: Table S6). Finally, the RT-qPCR experiments (four technical replicates) were performed for each of the selected lncRNA using OmicsGreen qPCR 5X Master Mix (Omics Bio) on a CFX96™ connect Real-Time PCR System (Bio-Rad). 1/100 of total converted cDNA was used as template cDNA for all RT-qPCR experiments, except for those shown in Fig. 1(b) in which 1/50 of total converted cDNA was used. In addition, for the experiments of whole bodies (Fig. 4), RT-qPCR experiments were also performed on three negative controls randomly picked up from un-transcribed regions (intergenic regions that are not expected to see any transcripts) for comparison.

### Availability of supporting data

The raw reads of brain samples have been submitted to NCBI Sequence Read Archive (SRA) database (SRP051132). The sequences and exon information of the curated 4599 lncRNAs were provided as Additional files 1 and 4.

## Additional files

Additional file 1:
**Sequences of the curated 4599 lncRNAs (FASTA format).** (FA 4819 kb) Additional file 2:
**Summary of the curated lncRNAs.** (XLS 1510 kb) Additional file 3: Figure S1. Distribution of lncRNA types in the different euchromatin regions. **Figure S2.** Occupied regions for each chromatin signature. **Table S1.** The length of lncRNAs. **Table S2.** RNA-seq datasets. **Table S3.** Statistics of exon numbers in lncRNA and mRNA genes from different sources. **Table S4.** Raw Ct values of RT-qPCR experiments for un-transcribed regions and the selected lncRNAs. **Table S5.** ChIP-seq datasets. **Table S6.** The primer list of the selected lncRNAs for RT-qPCR experiments. (PDF 356 kb) Additional file 4:
**Exon information of the curated lncRNAs (GFF format).** (GFF 878 kb) Additional file 5:
**Summary of novel lncRNAs discovery by the present study.** (XLS 142 kb)

## Abbreviations

ChIP-seq: Chromatin immunoprecipitation sequencing
CPC: Coding potential calculator
H3K4me3: Tri-methylation of H3 lysine 4
H3K36me3: Tri-methylation of H3 lysine 36
lincRNA: Long intergenic non-coding RNA
lncRNA: Long non-coding RNA
MSL: Male specific lethal
Pol II: RNA polymerase II
RT-qPCR: Quantitative reverse transcriptase-dependent polymerase chain reaction
TSS: transcription start site
