# Supplementary material for: Integrating RNA-seq and ChIP-seq data to characterize long non-coding RNAs in Drosophila melanogaster
Source: BMC Genomics. 2016 Mar 11;17:220. doi: 10.1186/s12864-016-2457-0 (PMC4787191; doi:10.1186/s12864-016-2457-0)
Supplement: Additional file 3: Figure S1. — Distribution of lncRNA types in the different euchromatin regions. Figure S2. Occupied regions for each chromatin signature. Table S1. The length of lncRNAs. Table S2. RNA-seq datasets. Table S3. Statistics of exon numbers in lncRNA and mRNA genes from different sources. Table S4. Raw Ct values of RT-qPCR experiments for un-transcribed regions and the selected lncRNAs. Table S5. ChIP-seq datasets. Table S6. The primer list of the selected lncRNAs for RT-qPCR experiments. (PDF 356 kb) [file 12864_2016_2457_MOESM3_ESM.pdf]

Integrating RNA-seq and ChIP-seq Data to Characterize Long Non-coding RNAs in *Drosophila melanogaster*

Mei-Ju May Chen[§], Li-Kai Chen[§], Yu-Shing Lai, You-Yu Lin, Dung-Chi Wu, Yi-An Tung, Kwei-Yan Liu, Hsueh-Tzu Shih, Yi-Jyun Chen, Yan-Liang Lin, Li-Ting Ma, Jian-Long Huang, Po-Chun Wu, Ming-Yi Hong, Fang-Hua Chu, June-Tai Wu[*], Wen-Hsiung Li[*] and Chien-Yu Chen[*]

[§]These authors contributed equally to this work

*Corresponding authors

SUPPLEMENTARY FIGURES AND TABLES

Figure. S1.	Distribution of lncRNA types in the different euchromatin regions.
Figure. S2.	Occupied regions for each chromatin signature.
Table S1.	The length of lncRNAs.
Table S2.	RNA-seq datasets.
Table S3.	Statistics of exon numbers in lncRNA and mRNA genes from different sources.
Table S4	Raw Ct values of RT-qPCR experiments for untranscribed regions and the selected lncRNAs
Table S5.	ChIP-seq datasets.
Table S6.	The primer list of the selected lncRNAs for RT-qPCR experiments.

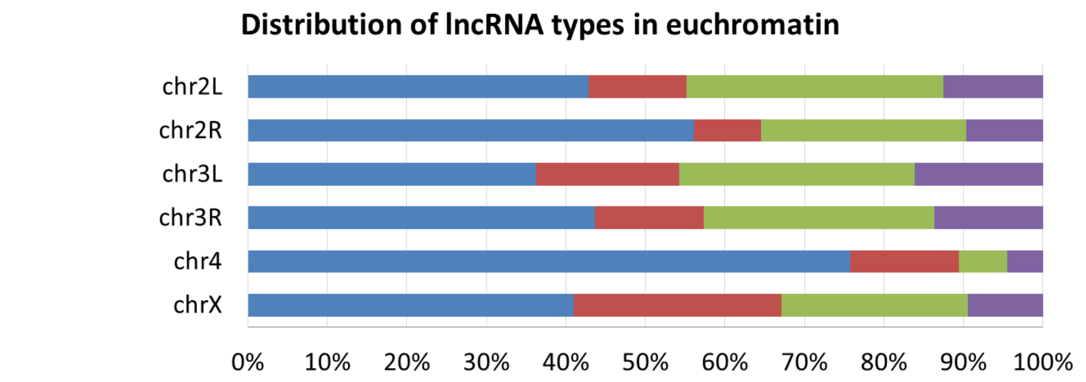

	chrX	chr4	chr3R	chr3L	chr2R	chr2L
anti-sense exonic	121	50	163	115	185	150
sense exonic	77	9	51	57	28	43
anti-sense intronic	69	4	108	94	85	113
sense intronic	28	3	51	51	32	44

Figure S1. Distribution of lncRNA types in euchromatin.

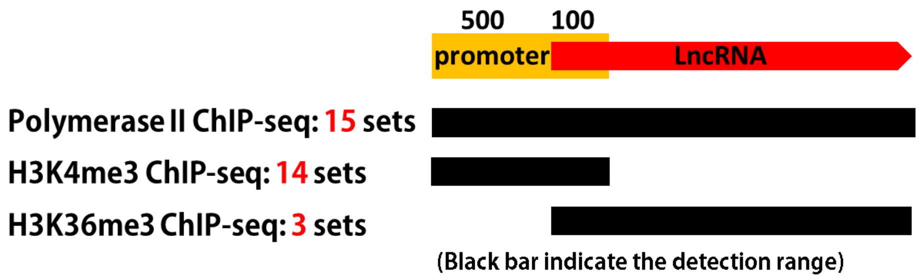

Figure S2. Occupied regions for each chromatin signature.

Table S1. The length of lncRNAs

Range	200~500	500~1000	1000~2000	2000~4000	4000~up	Total
FlyBase + UCSC	707	997	463	131	49	2347
Young et al.	189	179	130	60	25	583
Brown et al.	390	443	240	104	30	1207
Present study	130	214	93	23	2	462
Total	1416	1833	926	318	106	4599

Table S2. RNA-seq data sets

modENCODE ID / data generated by current study	Description	Usage in the present study
4433	Dm Dev Timecourse Expression RNA-seq AdF Ecl 1days sequences	Quantification for all of the curated lncRNAs / identification of exon-intron junction of lncRNAs from Young *et al*.
4434	Dm Dev Timecourse Expression RNA-seq AdF Ecl 30days sequences	Quantification for all of the curated lncRNAs / identification of exon-intron junction of lncRNAs from Young *et al*.
4435	Dm Dev Timecourse Expression RNA-seq AdF Ecl 5days sequences	Quantification for all of the curated lncRNAs / identification of exon-intron junction of lncRNAs from Young *et al*.
4436	Dm Dev Timecourse Expression RNA-seq AdM Ecl 1days sequences	Quantification for all of the curated lncRNAs / identification of exon-intron junction of lncRNAs from Young *et al*.
4437	Dm Dev Timecourse Expression RNA-seq AdM Ecl 30days sequences	Quantification for all of the curated lncRNAs / identification of exon-intron junction of lncRNAs from Young *et al*.
4438	Dm Dev Timecourse Expression RNA-seq AdM Ecl 5days sequences	Quantification for all of the curated lncRNAs / identification of exon-intron junction of lncRNAs from Young *et al*.
4439	Dm Dev Timecourse Expression RNA-seq em0-2h sequences	Quantification for all of the curated lncRNAs / identification of exon-intron junction of lncRNAs from Young *et al*.
4440	Dm Dev Timecourse Expression RNA-seq em10-12hr sequences	Quantification for all of the curated lncRNAs / identification of exon-intron junction of lncRNAs from Young *et al*.
4441	Dm Dev Timecourse Expression RNA-seq em12-14hr sequences	Quantification for all of the curated lncRNAs / identification of exon-intron junction of lncRNAs from Young *et al*.
4442	Dm Dev Timecourse Expression RNA-seq em14-16hr sequences	Quantification for all of the curated lncRNAs / identification of exon-intron junction of lncRNAs from Young *et al*.
4443	Dm Dev Timecourse Expression RNA-seq em16-18hr	Quantification for all of the curated

	sequences	lncRNAs / identification of exon-intron junction of lncRNAs from Young *et al*.
4444	Dm Dev Timecourse Expression RNA-seq em18-20hr sequences	Quantification for all of the curated lncRNAs / identification of exon-intron junction of lncRNAs from Young *et al*.
4445	Dm Dev Timecourse Expression RNA-seq em20-22hr sequences	Quantification for all of the curated lncRNAs / identification of exon-intron junction of lncRNAs from Young *et al*.
4446	Dm Dev Timecourse Expression RNA-seq em22-24hr sequences	Quantification for all of the curated lncRNAs / identification of exon-intron junction of lncRNAs from Young *et al*.
4447	Dm Dev Timecourse Expression RNA-seq em2-4hr sequences	Quantification for all of the curated lncRNAs / identification of exon-intron junction of lncRNAs from Young *et al*.
4448	Dm Dev Timecourse Expression RNA-seq em4-6hr sequences	Quantification for all of the curated lncRNAs / identification of exon-intron junction of lncRNAs from Young *et al*.
4449	Dm Dev Timecourse Expression RNA-seq em6-8hr sequences	Quantification for all of the curated lncRNAs / identification of exon-intron junction of lncRNAs from Young *et al*.
4450	Dm Dev Timecourse Expression RNA-seq em8-10hr sequences	Quantification for all of the curated lncRNAs / identification of exon-intron junction of lncRNAs from Young *et al*.
4451	Dm Dev Timecourse Expression RNA-seq L1 sequences	Quantification for all of the curated lncRNAs / identification of exon-intron junction of lncRNAs from Young *et al*.
4452	Dm Dev Timecourse Expression RNA-seq L2 sequences	Quantification for all of the curated lncRNAs / identification of exon-intron junction of lncRNAs from Young *et al*.
4453	Dm Dev Timecourse Expression RNA-seq L3 12hr sequences	Quantification for all of the curated lncRNAs / identification of exon-intron junction of lncRNAs from Young *et al*.
4454	Dm Dev Timecourse Expression RNA-seq L3 PS1-2 sequences	Quantification for all of the curated lncRNAs / identification of exon-intron junction of lncRNAs from Young *et al*.
4455	Dm Dev Timecourse Expression RNA-seq L3 PS3-6 sequences	Quantification for all of the curated lncRNAs / identification of exon-intron junction of lncRNAs from Young *et al*.
4456	Dm Dev Timecourse Expression RNA-seq L3 PS7-9 sequences	Quantification for all of the curated lncRNAs / identification of exon-intron junction of lncRNAs from Young *et al*.
4457	Dm Dev Timecourse Expression RNA-seq WPP 12hr sequences	Quantification for all of the curated lncRNAs / identification of exon-intron junction of lncRNAs from Young *et al*.
4458	Dm Dev Timecourse Expression RNA-seq WPP 24hr	Quantification for all of the curated lncRNAs / identification of exon-intron junc-

	sequences	tion of lncRNAs from Young *et al*.
4459	Dm Dev Timecourse Expression RNA-seq WPP 2days sequences	Quantification for all of the curated lncRNAs / identification of exon-intron junction of lncRNAs from Young *et al*.
4460	Dm Dev Timecourse Expression RNA-seq WPP 3days sequences	Quantification for all of the curated lncRNAs / identification of exon-intron junction of lncRNAs from Young *et al*.
4461	Dm Dev Timecourse Expression RNA-seq WPP 4days sequences	Quantification for all of the curated lncRNAs / identification of exon-intron junction of lncRNAs from Young *et al*.
4462	Dm Dev Timecourse Expression RNA-seq WPP sequences	Quantification for all of the curated lncRNAs / identification of exon-intron junction of lncRNAs from Young *et al*.
4291	Dm Tissue Expression RNA-seq mated female eclosion + 1 day heads multiply mapping reads	Identification of transcriptional directionality
4292	Dm Tissue Expression RNA-seq mated female eclosion + 20 days heads multiply mapping reads	Identification of transcriptional directionality
4293	Dm Tissue Expression RNA-seq mated female eclosion + 4 days heads multiply mapping reads	Identification of transcriptional directionality
4294	Dm Tissue Expression RNA-seq mated female eclosion + 4 days ovaries multiply mapping reads	Identification of transcriptional directionality
4295	Dm Tissue Expression RNA-seq mated male eclosion + 1 day heads multiply mapping reads	Identification of transcriptional directionality
4296	Dm Tissue Expression RNA-seq mated male eclosion + 20 days heads multiply mapping reads	Identification of transcriptional directionality
4297	Dm Tissue Expression RNA-seq mated male eclosion + 4 days accessory glands multiply mapping reads	Identification of transcriptional directionality
4298	Dm Tissue Expression RNA-seq mated male eclosion + 4 days heads multiply mapping reads	Identification of transcriptional directionality
4299	Dm Tissue Expression RNA-seq mated male eclosion + 4 days testes multiply mapping reads	Identification of transcriptional directionality
4300	Dm Tissue Expression RNA-seq mixed males and females eclosion + 1 day carcass multiply mapping reads	Identification of transcriptional directionality
4301	Dm Tissue Expression RNA-seq mixed males and females eclosion + 1 day digestive system multiply mapping reads	Identification of transcriptional directionality
4302	Dm Tissue Expression RNA-seq mixed males and females eclosion + 20 days carcass multiply mapping reads	Identification of transcriptional directionality
4303	Dm Tissue Expression RNA-seq mixed males and females eclosion + 20 days digestive system multiply mapping reads	Identification of transcriptional directionality
4304	Dm Tissue Expression RNA-seq mixed males and females eclosion + 4 days carcass multiply mapping reads	Identification of transcriptional directionality
4305	Dm Tissue Expression RNA-seq mixed males and females eclosion + 4 days digestive system multiply map-	Identification of transcriptional directionality

	ping reads	
4306	Dm Tissue Expression RNA-seq Pupae WPP+2d CNS multiply mapping reads	Identification of transcriptional directionality
4307	Dm Tissue Expression RNA-seq third instar larvae CNS multiply mapping reads	Identification of transcriptional directionality
4308	Dm Tissue Expression RNA-seq third instar larvae wandering stage carcass multiply mapping reads	Identification of transcriptional directionality
4309	Dm Tissue Expression RNA-seq third instar larvae wandering stage digestive system multiply mapping reads	Identification of transcriptional directionality
4310	Dm Tissue Expression RNA-seq third instar larvae wandering stage fat body multiply mapping reads	Identification of transcriptional directionality
4311	Dm Tissue Expression RNA-seq third instar larvae wandering stage imaginal discs multiply mapping reads	Identification of transcriptional directionality
4312	Dm Tissue Expression RNA-seq third instar larvae wandering stage salivary glands multiply mapping reads	Identification of transcriptional directionality
4313	Dm Tissue Expression RNA-seq virgin female eclosion + 1 day heads multiply mapping reads	Identification of transcriptional directionality
4314	Dm Tissue Expression RNA-seq virgin female eclosion + 20 days heads multiply mapping reads	Identification of transcriptional directionality
4315	Dm Tissue Expression RNA-seq virgin female eclosion + 4 days heads multiply mapping reads	Identification of transcriptional directionality
4316	Dm Tissue Expression RNA-seq virgin female eclosion + 4 days ovaries multiply mapping reads	Identification of transcriptional directionality
4317	Dm Tissue Expression RNA-seq WPP+2d fat multiply mapping reads	Identification of transcriptional directionality
4318	Dm Tissue Expression RNA-seq WPP fat body multiply mapping reads	Identification of transcriptional directionality
4319	Dm Tissue Expression RNA-seq WPP salivary glands multiply mapping reads	Identification of transcriptional directionality
Current study (SRP051132)	Dm four-day post-eclosion wild-type male brains with poly(A)-enriched library	Discovery of novel lncRNA
Current study (SRP051132)	Dm four-day post-eclosion wild-type male brains with Ribo-Zero library	Discovery of novel lncRNA

Table S3. Statistics of exon numbers in lncRNA and mRNA genes from different sources.

Exon num.	FlyBase + UCSC	Young et al.	Brown et al.	Present study	mRNA
1	1167	444	465	422	2751
2	495	93	163	33	4739
3	196	32	60	6	4109
4	68	12	35	1	3659
5	36	1	15	0	2863
6	17	0	8	0	2268
7	8	0	7	0	2003
8	2	1	7	0	1586
9	3	0	2	0	1281
10	1	0	2	0	995
11	1	0	5	0	781
12	3	0	0	0	612
13	0	0	0	0	471
14	0	0	0	0	391
15	0	0	1	0	331
16	0	0	0	0	240
17	0	0	0	0	200
18	1	0	0	0	145
>=19	1	0	2	0	837
Total	1999	583	772	462	30262

Table S4. Raw Ct values of RT-qPCR experiments for un-transcribed regions and the selected lncRNAs

	RT+				RT-			
**Replicates**	**P1**	**P2**	**P3**	**P4**	**N1**	**N2**	**N3**	**N4**
**Figure 1(a) RT-qPCR experiments for a selected set of lncRNAs in brains**								
RpL32	21.89	21.92	22.04	21.97	35.73	35.23	35.29	35.55
ROX1	20.33	20.15	20.33	20.35	32.11	32.31	32.2	32.3
TCONS_00031380	26.67	26.47	26.46	26.57	32.55	32.87	32.77	NA
TCONS_00028095	26.37	26.32	26.24	26.11	NA	31.99	32.16	32.13
TCONS_00044977	28.07	27.98	27.97	28.12	NA	33.42	33.35	33.49
TCONS_00037494	NA	28.86	28.7	28.78	33.32	33.27	33.18	33.18
TCONS_00048859	28.29	28.37	28.14	27.91	32.32	32.24	32.02	31.72
TCONS_00051944	NA	33.7	33.46	33.77	37.43	37.08	36.58	36.45
TCONS_00045108	27.42	27.29	27.41	27.42	30.31	30.22	30.25	NA
TCONS_00020613	29.42	29.22	29.27	29.2	32.08	31.91	31.81	31.78
TCONS_00033121	31.7	31.57	31.47	31.39	34.13	34.05	33.47	33.73
TCONS_00017414	29.55	29.21	29.29	29.2	NA	31.6	31.62	31.55
TCONS_00050427	30.88	30.91	30.91	31.06	33.09	33.05	32.65	33.03
TCONS_00032409	30.43	30.35	30.18	30.16	NA	32.21	31.77	31.86
TCONS_00036092	30.25	30.12	29.75	29.61	31.62	31.73	31.52	31.14
TCONS_00044754	30.37	30.51	30.55	30.55	31.94	32.04	32.09	32.06
TCONS_00003446	31.42	31.65	31.32	31.36	32.68	32.88	33.03	32.88
TCONS_00043412	31.96	31.97	31.94	32.12	33.39	33.3	33.17	33.47
TCONS_00020772	26.1	25.95	26.08	26.16	27.42	26.93	26.87	27.15
TCONS_00036539	32.12	32.24	32.04	32.22	32.66	33.25	33.02	32.85
TCONS_00044991	31.11	31	31.01	31.06	31.58	31.42	31.76	NA
TCONS_00044992	NA	31.07	31.25	31.14	31.67	31.66	31.6	31.73
TCONS_00034204	31.28	31.24	31.15	31.17	31.93	31.68	31.6	31.5
TCONS_00011851	30.98	30.8	30.72	30.56	30.98	30.8	30.72	30.56
**Figure 1(b) RT-qPCR experiments for a selected set of lncRNAs in brains: 2-fold amount of template brain cDNA**								
RpL32	21.52	21.45	21.57	21.68	35.49	35.02	36.25	35.31
ROX1	20.03	19.79	19.86	19.65	33.18	33.08	33.18	33.27
FBgn0051144	27.26	27.05	27.07	26.92	33.16	33.65	32.63	32.94
FBgn0265590	NA	25.78	25.89	25.7	NA	31.33	31.55	31.48
FBgn0262107	25.58	25.62	25.62	25.66	31	30.98	31.13	31.04
FBgn0264360	30.27	30.19	30.11	30.16	NA	32.76	32.75	32.63
FBgn0266811	29.72	29.52	NA	29.72	31.42	31.26	30.33	30.66
FBgn0267298	32.68	32.53	32.62	NA	33.63	33.67	33.51	NA
FBgn0264980	31.32	31.39	31.39	NA	31.79	31.79	31.73	31.97
FBgn0264993	32.28	32.45	32.16	32.18	32.27	32.18	32.2	32.38
FBgn0263331	31.35	31.38	31.29	31.46	31.39	31.58	31.25	31.42

TCONS_00036539	31.91	32.23	32.02	32.26	32.98	32.66	32.92	33
TCONS_00044991	31.22	31.14	31.04	30.81	32.43	32.28	32.31	32.03
TCONS_00044992	31.05	31	30.84	30.83	NA	32.02	32.04	31.96
TCONS_00034204	31.37	31.26	31.43	31.23	32.17	32.2	31.79	32.02
TCONS_00011851	30.77	30.66	30.48	30.76	32.99	33.02	32.57	32.41

Figure 4. RT-qPCR experiments of a selected set of lncRNAs in male adults

Untranscribed_region1	33.19	33.17	33.43	33.38	33.40	33.40	33.33	33.36
Untranscribed_region2	33.40	33.89	33.55	33.73	33.79	33.58	34.02	33.80
Untranscribed_region3	33.20	33.19	33.19	33.21	33.15	33.13	33.26	33.16
G1_FBgn0083068	26.67	26.57	26.49	26.55	35.70	36.12	36.52	35.80
G1_FBgn0265590	27.39	27.39	27.36	27.41	35.17	35.13	34.18	35.32
G1_TCONS_00045108	29.33	29.36	29.08	29.11	34.20	35.07	34.81	35.13
G1_FBgn0001234	22.68	22.64	22.61	22.70	36.02	36.21	35.40	36.33
G1_FBgn0051144	27.12	27.14	27.01	27.04	33.54	NA	35.29	35.27
G1_FBgn0262109	26.91	27.03	26.80	27.04	NA	40.62	NA	39.78
G1_FBgn0264360	24.28	24.27	24.27	24.20	31.24	31.08	31.19	31.26
G1_FBgn0265071	26.47	26.46	26.42	26.35	32.25	33.23	NA	31.69
G1_FBgn0265295	26.19	26.24	26.12	26.18	34.28	34.07	35.24	34.25
G1_ROX1	23.62	23.57	23.47	23.56	38.14	NA	38.20	38.48
G1_ROX2	28.44	28.49	28.31	NA	37.70	NA	36.31	36.76
G2_FBgn0263981	26.79	26.69	26.57	26.53	37.53	NA	36.05	38.07
G2_FBgn0264869	32.12	31.71	31.70	31.69	37.19	36.72	36.58	36.05
G2_FBgn0262993	32.59	32.39	32.46	32.64	34.35	35.70	34.44	35.21
G2_FBgn0265340	29.58	29.62	29.35	29.53	33.52	33.80	33.72	34.24
G2_FBgn0260720	28.58	28.40	28.18	28.29	32.72	33.23	33.36	33.51
G2_TCONS_00012337	28.56	28.28	28.27	28.25	32.93	33.54	33.21	33.71
G2_lincRNA.292	21.14	21.14	21.21	21.19	35.42	34.48	34.40	34.66
G2_FBgn0264446	31.07	NA	31.09	31.04	32.59	31.56	32.22	32.41
G2_FBgn0264481	NA	30.00	29.83	30.00	NA	35.93	35.91	36.01
G2_FBgn0264504	29.99	NA	29.74	29.89	33.03	NA	32.32	32.99
G2_FBgn0266044	27.35	NA	27.25	27.29	32.93	33.33	NA	33.76
G3_FBgn0264993	27.11	27.17	27.06	27.04	36.93	36.59	35.89	37.22
G3_FBgn0265458	28.25	28.20	28.13	28.09	35.75	34.81	35.47	35.54
G3_TCONS_00045565	13.39	13.46	13.39	13.34	25.78	25.77	25.96	25.90
G3_FBgn0262106	26.11	26.08	25.98	26.10	35.99	35.42	36.61	37.08
G3_FBgn0262107	27.02	26.89	26.67	26.76	35.96	35.01	35.10	35.01
G3_FBgn0264980	27.40	27.47	27.48	27.45	36.72	35.26	35.48	35.57
G3_FBgn0062928	25.30	25.24	25.18	25.11	34.47	34.03	34.03	34.25
G3_lincRNA.354	26.06	26.06	25.89	25.89	32.63	32.63	33.71	32.48
G3_FBgn0263331	26.48	26.45	26.46	26.42	33.08	33.20	32.47	32.17
G3_FBgn0263626	24.52	24.46	24.37	NA	27.95	27.83	27.97	28.16
G4_FBgn0265530	31.40	31.54	31.22	31.31	35.44	35.79	35.38	34.41

G4_TCONS_00054835	33.21	33.11	33.29	31.13	33.53	34.73	34.39	33.04
G4_lincRNA.160	27.14	27.10	27.02	28.53	36.44	35.51	35.98	35.52
G4_FBgn0263380	33.05	32.41	32.27	32.47	34.90	35.78	35.34	35.25
G4_FBgn0264840	28.17	28.33	28.24	28.29	35.99	36.14	NA	36.79
G4_FBgn0265302	34.08	34.29	34.19	NA	40.50	40.42	40.24	40.37
G4_TCONS_00020772	26.24	26.21	26.00	26.11	28.26	28.29	28.28	28.18
G4_FBgn0263497	31.50	31.62	31.35	NA	33.71	34.30	33.46	33.99
G4_FBgn0262963	NA	31.82	31.68	31.62	35.14	34.11	34.12	35.19
G4_FBgn0265085	29.31	29.38	29.26	29.38	35.02	35.57	34.57	36.25

Table S5. ChIP-seq data sets

modENCODE ID	Chromatin signature	Stage	Reference
4950	H3K36me3	Embryos 14-16 hr	Karpen, G.
4941	H3K36me3	Larvae 3rd instar	Karpen, G.
5091	H3K36me3	Mixed Adult	Karpen, G.
5096	H3K4me3	Embryos 14-16 hr	Karpen, G.
5098	H3K4me3	Mixed Adult	Karpen, G.
789	H3K4me3	Embryos 0-4 hr	White, K.
790	H3K4me3	Embryos 4-8 hr	White, K.
791	H3K4me3	Embryos 8-12 hr	White, K.
792	H3K4me3	Embryos 12-16 hr	White, K.
793	H3K4me3	Embryos 16-20 hr	White, K.
794	H3K4me3	Embryos 20-24 hr	White, K.
795	H3K4me3	Larvae L1 stage	White, K.
796	H3K4me3	Larvae L2 stage	White, K.
797	H3K4me3	Larvae L3 stage	White, K.
798	H3K4me3	Pupae	White, K.
799	H3K4me3	Adult Female	White, K.
800	H3K4me3	Adult Male	White, K.
984	RNA polymerase II	Larvae L2 stage	White, K.
850	RNA polymerase II	Embryos 20-24 hr	White, K.
4193	RNA polymerase II	Embryos 4-7 hr	MacAlpine, D.
3251	RNA polymerase II	Embryos mixed stages 0-24 hr	MacAlpine, D.
3625	RNA polymerase II	Embryos 0-2 hr	MacAlpine, D.
848	RNA polymerase II	Embryos 12-16 hr	White, K.
4352	RNA polymerase II	Larvae 3rd instar	MacAlpine, D.
5122	RNA polymerase II	Embryos 14-16 hr	Karpen, G.
5123	RNA polymerase II	Larvae 3rd instar	Karpen, G.
5124	RNA polymerase II	Mixed Adult	Karpen, G.
846	RNA polymerase II	Embryos 4-8 hr	White, K.
847	RNA polymerase II	Embryos 8-12 hr	White, K.
851	RNA polymerase II	Larvae L1 stage	White, K.
852	RNA polymerase II	Larvae L3 stage	White, K.
853	RNA polymerase II	Pupae	White, K.

Table S6. Primer list of the selected lncRNAs for RT-qPCR experiments

ID	5' primer	3' primer	Experiment results
TCONS_00031380	AGTCCTTCGAAACAAACTGTCT	TTGGTAAACAATGCGGCAATAC	Figure 1(a)
TCONS_00028095	ATACATTGTGCCAAAATAGCCG	AATTCACAGCCCTTCTTAGCAT	Figure 1(a)
TCONS_00044977	TCGATGATTCTACGGTCAAGTT	TTTTTGTTTGCCGAACATCTCG	Figure 1(a)
TCONS_00037494	AGCCTATGGACAAGGACATCTA	TATGATGTGTAATTGGTCGGCA	Figure 1(a)
TCONS_00048859	CCACTTAAAGGAGGCGATCTTC	AAGATGCTGAGGATATGGATGC	Figure 1(a)
TCONS_00051944	ATCCGGATATTCGACCTTGTTG	ATTTTAGTTGCGCTTGCTGTTC	Figure 1(a)
TCONS_00020613	GAAAAGGCAGCAAGTGTTACAA	ACCAAACTGCTGGTATCGTTAT	Figure 1(a)
TCONS_00033121	GCTTCGATCATTTCGCGTATC	CCACTAGCGATGATGGTGAAAG	Figure 1(a)
TCONS_00017414	TCGCTGACGACAAAATCCTTAT	TACGTTTACTTTTCGTGAGGCT	Figure 1(a)
TCONS_00050427	ATCCAGATGCCAGAATTCACC	ATGTGGATGTGACCTGAATCAC	Figure 1(a)
TCONS_00032409	GTGTCGTGCTACATGTGTTTAC	GAGAAGAAAACAAGGTGCTGTG	Figure 1(a)
TCONS_00036092	ATTTCCATTGTTGTTGCCATGC	CGGCGGTCCAATACAAACAATA	Figure 1(a)
TCONS_00044754	GGAACTAGGGGCATTTAGTTGT	CAACATATGCGGAGGGATTTTG	Figure 1(a)
TCONS_00003446	TCTTGGGCTGAGAATAATGCAA	ATATTCCAACAGCCCACTAACG	Figure 1(a)
TCONS_00043412	CATGGCTACTCACTCAGGTAGA	CTAATGGCTTCTTGATGCGTTC	Figure 1(a)
TCONS_00036539	ACCAACTCGGCAACAACTATAA	CTTACAGTTGCACGACAACAAC	Figure 1
TCONS_00044991	AATCGTTACACTAAACACCCGA	ACTCGCTACACATCCCTAAGTA	Figure 1
TCONS_00044992	TGACGACACATAGCTGAAAAGT	CAGAAGCTCAAGCAAATTCCTC	Figure 1
TCONS_00034204	CAGCTTGAATTGGGTCAAGTTT	CACACCAGCTGACAGTTATTTC	Figure 1
TCONS_00011851	GAACGGAACCGCAAAACTAAG	CTGCCCTTTGATGCTAAATGTC	Figure 1
FBgn0266811	TCATAATGGAACTATGCAGGCG	ATTTCAATACGTTTAGGCACGC	Figure 1(b)
FBgn0267298	AAACACTTGAAATGGACTTGGC	TGTTCGGGTATCCTCGCTAAAT	Figure 1(b)
Untranscribed_region1	ACTCTCGTAGAAACAATCTCGT	GCAAAAGTTAAAAGGACACAGC	Figure 4
Untranscribed_region2	CGCATTTATTATGCCATCCTCA	GTATTGATGCCGGTGTACTTTT	Figure 4
Untranscribed_region3	ATCACACGATAACAACAAAGGG	CTCCTCCGATGATTTTAGTCCT	Figure 4
G1_FBgn0083068	ATCGGACGGAAATGCAGAAG	CACTGGGAGGGCTAATGAAC	Figure 4
G1_FBgn0265590	CAAGAAGTGGAAGGGAGATGG	GACAGGCGCAACAACTAAAC	Figure 1(b) and Figure 4
G1_TCONS_00045108	CTAACCAGACGCTCTCAGTC	CCCCTCCCTTCAAACAAGATAC	Figure 1(a) and Figure 4
G1_FBgn0001234	CACTGGTGTATCGACTTCTCTG	GTATGTCTGCCCTTTACGGAAC	Figure 4
G1_FBgn0051144	CTAAGAGGCCGATCAGAAGG	CTTCCTACTCCATTTGTCGC	Figure 1(b) and Figure 4
G1_FBgn0262109	TCGTAAAGGGAATCCAACGC	GATGCAATCGTCAGCGAAGTC	Figure 4
G1_FBgn0264360	ATATGCTGCTCTGCGTCTTC	TCTGTTTACGTGTTGGCGTC	Figure 1(b) and Figure 4
G1_FBgn0265071	CTTCTTCTTGCTACCCGCTTTG	TCTGCTCATAATTGCGCTCG	Figure 4
G1_FBgn0265295	GTAGTAGACGTGAGCCAAGTTC	GTTGGAGGTGCCCACAATTATC	Figure 4
G1_ROX1	ACATCAGGCCATAGCCAAGAAG	AACACGATCTACTTCTGGTCGG	Figure 1 and Figure 4
G1_ROX2	GGTCACACTAAGCTAGGGCTAC	CGGAAATCGTTACTCTTGCTTG	Figure 4
G2_FBgn0263981	CAGCTCCAGCATTTCCTTAACC	CGTACAGCTTATCCATATCGGC	Figure 4
G2_FBgn0264869	CTCGACTCAACACAATTCCGAC	CAACACGAGGTATGTTTCTCCC	Figure 4
G2_FBgn0262993	GGACAACCATAGAATGAGGGAG	CGAATGCGAGAAAGAGAGGTAG	Figure 4

G2_FBgn0265340	CCCAACCATTGATGAAGCTGTG	GTATAGTCTAACGGCGGAGATG	Figure 4
G2_FBgn0260720	CCATCACCATCTTCAATAGCCC	TGCTACATAAGCCAGTCAGTG	Figure 4
G2_TCONS_00012337	ATTTCAAGTTGCCCCCAGTC	CTCGATTTCAGGCCAAGAGAG	Figure 4
G2_lincRNA.292	CCTTCTGATAACCCTTGTGGC	GCTGATAGATACGGAAGTGGTC	Figure 4
G2_FBgn0264446	TACCTTCGCATCACTGCTTC	GGATTTGGGTTTTGGGCTTG	Figure 4
G2_FBgn0264481	CGTCATTCTCTTCCTCCGATG	GTCGTGTCTGTGTGTGCTTA	Figure 4
G2_FBgn0264504	CAAAGACTGTTCCTGCTCCTG	CCATGTTCCCAGCTTACGATTG	Figure 4
G2_FBgn0266044	GGAGTGAGTTAAGGGACAACAG	CGCTGCTGAGATTGGAGTTAG	Figure 4
G3_FBgn0264993	CTTCGATGAGCACCAGGATAC	CATGGGATTCAAGTACGACAGC	Figure 1(b) and Figure 4
G3_FBgn0265458	CCCCAATGTCTTCGACTTACTC	CAGGAGGATCTGTTTCTGGAC	Figure 4
G3_TCONS_00045565	AGTCTAACCTGCCCACTGAA	CCAACCATTCATTCCAGCCTTC	Figure 4
G3_FBgn0262106	GTCATTCATACTGGGTCTTGCC	TCCATTTCGGGTTTGGTGAC	Figure 4
G3_FBgn0262107	ATGACCAAGAGGATGAGTCGC	GCTACTGCTGTCTATAAGGTGG	Figure 1(b) and Figure 4
G3_FBgn0264980	CTAATTTCACTCTACCCGCCG	CTCAACTCAACCGACCCTTAC	Figure 1(b) and Figure 4
G3_FBgn0062928	GAACCGAAAGCACCAGATCC	GGAGGAGAGTAAGCCACGTTAG	Figure 4
G3_lincRNA.354	GTGGCTATAATGATCCCGGTAG	GTGATGATCTCCCATTCTCTGC	Figure 4
G3_FBgn0263331	CGCTTGTGGGTGAAGCATTG	TGCCGCCAGAATGAGATTCC	Figure 1(b) and Figure 4
G3_FBgn0263626	CTCTACCCCATCCATTTTCAGG	CTGTGTGCTCTGTTATGTGTCC	Figure 4
G4_FBgn0265530	CGAATCAACCAGACCCATAAGC	TGGCGATATTTGACAGACGG	Figure 4
G4_TCONS_00054835	CCCATTATCCTCTGCAAGTGTG	GAGAGTCGGAAATCGAGAATCG	Figure 4
G4_lincRNA.160	GTATGAAAAAGTGGAGCGACGG	CCCACCATCCCCTAAACAAAG	Figure 4
G4_FBgn0263380	CAATCATGGAGATGGAGGACC	CGGAGTCTTCAGTTCGAGTTC	Figure 4
G4_FBgn0264840	AAGACAGGTTAAGGCTAGTCGG	CTCATGCCGAAACACATTCG	Figure 4
G4_FBgn0265302	GCCTTCTCCAGTTTGGTATGAC	ACAATTAGCCCGACCATCTC	Figure 4
G4_TCONS_00020772	GAGTGGATAGCGGAGATTGC	GCCTTCTTGACTTCCTTCTCC	Figure 1(a) and Figure 4
G4_FBgn0263497	ATCGAATCGGTGGTAAGTGAGG	GGAAAGTGAGCGGGTTAAAGTG	Figure 4
G4_FBgn0262963	GTTCTGGGGTCAGTTGGACT	AACCAAAGAGGGAAATGCGG	Figure 4
G4_FBgn0265085	CATCTGAACCCCAACCACTTC	GAGCACAAGCACCAACAATG	Figure 4
